# An allelic atlas of immunoglobulin heavy chain variable regions reveals antibody binding epitope preference resilient to SARS-CoV-2 mutation escape

**DOI:** 10.3389/fimmu.2024.1471396

**Published:** 2025-01-07

**Authors:** Weiqi Deng, Xuefeng Niu, Ping He, Qihong Yan, Huan Liang, Yongping Wang, Lishan Ning, Zihan Lin, Yudi Zhang, Xinwei Zhao, Liqiang Feng, Linbing Qu, Ling Chen

**Affiliations:** ^1^ State Key Laboratory of Respiratory Disease, Guangdong Laboratory of Computational Biomedicine, Center for Cell Lineage Research, Guangzhou Institutes of Biomedicine and Health, Chinese Academy of Sciences, Guangzhou, China; ^2^ University of Chinese Academy of Science, Beijing, China; ^3^ State Key Laboratory of Respiratory Disease, Guangzhou Institute of Respiratory Health, the First Affiliated Hospital of Guangzhou Medical University, Guangzhou, China; ^4^ Guangzhou National Laboratory, Guangzhou, China

**Keywords:** allele, affinity, antibody, epitopes, immunoglobulin heavy chain (Igh), neutralization, receptor-binding domain (RBD), SARS-CoV-2

## Abstract

**Background:**

Although immunoglobulin (Ig) alleles play a pivotal role in the antibody response to pathogens, research to understand their role in the humoral immune response is still limited.

**Methods:**

We retrieved the germline sequences for the IGHV from the IMGT database to illustrate the amino acid polymorphism present within germline sequences of IGHV genes. We aassembled the sequences of IgM and IgD repertoire from 130 people to investigate the genetic variations in the population. A dataset comprising 10,643 SARS-CoV-2 spike-specific antibodies, obtained from COV-AbDab, was compiled to assess the impact of SARS-CoV-2 infection on allelic gene utilization. Binding affinity and neutralizing activity were determined using bio-layer interferometry and pseudovirus neutralization assays. Primary docking was performed using ZDOCK (3.0.2) to generate the initial conformation of the antigen-antibody complex, followed by simulations of the complete conformations using Rosetta SnugDock software. The original and simulated structural conformations were visualized and presented using ChimeraX (v1.5).

**Results:**

We present an allelic atlas of immunoglobulin heavy chain (IgH) variable regions, illustrating the diversity of allelic variants across 33 IGHV family germline sequences by sequencing the IgH repertoire of in the population. Our comprehensive analysis of SARS-CoV-2 spike-specific antibodies revealed the preferential use of specific Ig alleles among these antibodies. We observed an association between Ig alleles and antibody binding epitopes. Different allelic genotypes binding to the same RBD epitope on the spike show different neutralizing potency and breadth. We found that antibodies carrying the IGHV1-69*02 allele tended to bind to the RBD E2.2 epitope. The antibodies carrying G50 and L55 amino acid residues exhibit potential enhancements in binding affinity and neutralizing potency to SARS-CoV-2 variants containing the L452R mutation on RBD, whereas R50 and F55 amino acid residues tend to have reduced binding affinity and neutralizing potency. IGHV2-5*02 antibodies using the D56 allele bind to the RBD D2 epitope with greater binding and neutralizing potency due to the interaction between D56 on HCDR2 and K444 on RBD of most Omicron subvariants. In contrast, IGHV2-5*01 antibodies using the N56 allele show increased binding resistance to the K444T mutation on RBD.

**Discussion:**

This study provides valuable insights into humoral immune responses from the perspective of Ig alleles and population genetics. These findings underscore the importance of Ig alleles in vaccine design and therapeutic antibody development.

## Introduction

Since 2019, SARS-CoV-2 has triggered a relentless global pandemic, posing a severe threat to human health. As of December 30, 2023, there have been more than seven billion confirmed cases of severe coronavirus disease 2019 (COVID-19) worldwide, with nearly seven million cumulative deaths (https://covid19.who.int). Global vaccination campaigns have been carried out to protect public health, aiming to induce adaptive immune responses in individuals to defend against viral invasion. However, not all vaccine recipients can generate a robust adaptive immune response. Some individuals may not attain sufficient protection after the first dose and need multiple doses to boost their humoral immunity against the virus (1–3). With the continuous mutation of SARS-CoV-2 and the emergence of new variants, varying degrees of decline in humoral immune responses have been observed in vaccinated individuals (4–7). This suggests that different people have varying degrees of tolerance to mutated strains. Yet, the mechanisms leading to this phenomenon remain unclear.

Humoral immunity, a vital component of the adaptive immune response, relies on B lymphocytes producing specific antibodies to protest pathogenic infections (6). The diversity of antibodies is shaped through processes like V(D)J recombination, allelic variations, and somatic hypermutation, allowing the body to generate numerous unique antibodies that confer protection through various molecular mechanisms (8–10). These unique antibodies collectively form the antibody repertoire. Allelic variations refer to genetic diversity in the open reading frame encoding antibodies, particularly in the variable areas (9, 11). Unlike more random events such as somatic hypermutation, allelic variations consistently generate a diverse population of antibodies. In humans, heavy chain genes on chromosome 14 consist of 129 variable genes (V), 27 diversity genes (D), and 9 joining genes (J). Notably, heavy chain allelic loci play a vital role, constituting 62.5% (267/427) of antibody allelic loci and underscoring their significance in forming antibodies (9).

However, there are limited studies on the influence of antibody allelic genes and their roles in responding to infections. For instance, it has been reported that in the influenza vaccinees, F55 of IGHV1-69 plays a crucial role in forming broad-spectrum antibodies against H5N1 (12). Another study showed that F55 is essential for the initial development of the IGHV1-69 antibodies binding the influenza H1-HA stem region, playing a crucial role in initiating the antibody affinity maturation process (13). During the MERS-CoV outbreak, a MERS-CoV neutralizing antibody m336, utilizing IGHV1-69*06, was identified. It was found that F55 in HCDR2 and K74 in FR3 significantly impact the high affinity for the spike (14). Since the outbreak of the SARS-CoV-2 pandemic, there have been emerging reports on the functional analysis of allelic variants. An earlier study showed that a class of RBD-specific antibodies, represented by LY-CoV1404, utilizes the IGHV2-5*02 genotype containing D56. These antibodies have broad-spectrum neutralizing activities against WT, Delta, and early Omicron subvariant BA.2. In contrast, IGHV2-5*01, which contains N56 is absent in IGHV2-5/IGLV2-14-encoded RBD monoclonal antibodies (15). CAB-I47, an antibody utilizing the IGHV1-69*20 genotype, with R50 and F55 amino acid residues in the CDR2 region, can effectively neutralize WT strain. However, the use of G50 and L55 amino acid residues completely abolishes both binding and neutralizing activity (16). Another study showed that both F55 and L55 alleles encode broadly neutralizing antibodies (bnAbs) against the same epitope in the human influenza virus. However, while humanized transgenic mice carrying F55/F55 and F55/L55 genotypes could generate bnAbs, those carrying the L55/L55 genotype could not (17). A computational analysis using the Structural Antibody Database (SAbDab; http://opig.stats.ox.ac.uk/webapps/sabdab) (18) suggested that polymorphisms in 73% of the alleles may affect antibody binding activity (19). Despite these reports on antibody genotypic variations, the lack of systematic research on population genotypes has constrained our understanding of humoral immunity.

This study aimed to provide an overview of human antibody heavy chain region allelic genes and to construct an allelic atlas of IgH variable regions. We applied immunoglobulin heavy chain repertoire sequencing to display the distribution of allelic variants in the population. Using SARS-CoV-2 as a model, we performed analysis on approximately 10,000 spike-specific antibodies in public databases, attempting to summarize their allelic gene usages and binding epitope preferences. Finally, we selected two representative antibody classes, IGHV1-69 and IGHV2-5, and delved into the impact of allelic genes on the binding and neutralizing activities.

## Materials and methods

### Research cohort and bulk immunoglobulin heavy chain gene sequencing

IgH sequencing technology is a high throughput method for analyzing immunoglobulin heavy chain gene rearrangement and diversity (20). IgH sequencing data from 130 people, consisting of 106 COVID-19 convalescents and 24 healthy individuals, were used in this study, which has been described in previous reports (21–23). All individuals are Asian. The median age of healthy individuals is 44.5 years (range 24-68), while the median age of COVID-19 convalescents is 57.1 years (range 25-87). In terms of gender distribution, men comprise 41.7% (10/24) of the healthy individuals and 39.6% (42/106) of the COVID-19 individuals. Conversely, women account for 58.3% (14/24) of healthy individuals, 60.4% (64/106) of COVID-19 convalescents. Peripheral blood mononuclear cells (PBMCs) from blood samples were isolated using Opti-Prep lymphocyte separation solution (Axis Shield Poc As, Norway) and subsequent centrifugation (24). Then, total RNA from the isolated cells was purified using TRIzol™ (ThermoFisher, USA) (25). Next, iRepertoire employed the damPCR technology with highly specific multiplex primers (iRepertoire, Inc., USA) to amplify high-purity BCR and TCR sequences. Finally, the obtained sequences were read using the Illumina HiSeq 2500 with a 2x250bp sequencing mode (Novogene, China) (23).

### Bioinformatics analysis

The raw data was initially subjected to filtering using Trimmomatic (v0.39) to clean sequences with a quality score under 20 (26). Subsequently, qualified paired-end sequencing reads were subjected to Flash (v1.2.11) assembly and converted into FASTA files (27). Finally, a reference index was constructed using germline sequences from IMGT, and MiXCR (v4.0) was employed to filter IgH sequences containing V(D)J information (28). The IgM and IgD repertoire data were used to identify allelic genotypes in the population through MiXCR analysis, with all reference sequences derived from IMGT reference sequences. The observed non-allelic variant amino acids are referred to as somatic hypermutation. And the criteria for “less prevalent” are as follows. 1. Functional antibody families with allelic sites in the sequencing region. 2. At least 200 reads per 100k reads for the antibody family. 3. The antibody family can be detected in more than 50% of individuals. The information on SARS-CoV-2 specific antibody sequences was obtained from the Coronavirus Antibody Database (https://opig.stats.ox.ac.uk/webapps/covabdab/) (29). NCBI IgBLAST (v1.18.0) was used to search and annotate the alleles for each antibody (30). The data on neutralizing antibodies against COVID-19 were sourced from published articles (31, 32). We utilized the epitope classification method from these articles and annotated each antibody’s allelic information for further analysis. The germline reference sequences are derived from IMGT/GENE-DB (Version 3.1.39), using the amino acid sequences from V-REGION with F+ORF+in-frame P including IMGT gaps, and IMGT gaps were removed in subsequent alignment analyses. The numbering used in our study is the IMGT unique numbering, excluding any gaps. Due to the high mutation rate of the CDR3 region, our study focused on 3 framework regions (FR1, FR2, FR3) and 2 complementarity determining regions (CDR1, CDR2).

### Monoclonal antibody expression and purification

Antibody heavy and light chain amino acid sequences were obtained from the Coronavirus Antibody Database (29). To avoid issues caused by unsuitable nucleotides, all sequences were synthesized according to human codon usage. The IGHV1-69 heavy chains were reverted to their IGHV1-69*02 germline sequences. The sequence of IGHV2-5 antibodies was not modified due to the significant functional changes caused by the D56 alteration. The plasmids were then transformed into DH5α cells (Escherichia coli) for large-scale amplification, and the plasmids carrying the heavy and light chains were extracted using an endotoxin-free plasmid extraction kit (Macherey-Nagel, Germany). The heavy and light chain plasmids, wrapped in equal amounts with PEI (Life-iLab Biotech), were transfected into Expi 293 cells (Thermo Fisher). Subsequently, protein A resin was employed for purification, resulting in high-concentration IgG1 antibodies against SARS-CoV-2 and their variants. All purified proteins were stored in PBS buffer (BasalMedia) and preserved at -80°C.

### BLI detection for neutralizing antibody affinity and binding kinetics

The SARS-CoV-2 specific antibodies and the RBD protein were prepared in advance and stored on ice. They were then diluted to 11 μg/mL and 200 mM using PBS-TB buffer (PBS with 0.02% v/v Tween-20 and 0.1% w/v BSA) separately. The AHC2 biosensor (Sartorius) was initially pre-wetted with PBS-TB buffer for ten minutes and loaded with antibodies until the signal response reached approximately 1.5 nm. Subsequently, the antibodies were exposed to various RBD solutions for 300 seconds to evaluate the association kinetics. Finally, the biosensor was placed in a PBS-TB buffer for 600 seconds to assess the dissociation kinetics. All procedures were conducted at 25°C with an orbital shaking speed of 1,000 rpm. Binding response less than 0.1 was considered as non-binding.

### Pseudovirus neutralization assay

The WT, BQ.1.1, and XBB pseudoviruses were primarily constructed using lentivirus as the main backbone, each carrying different subtypes of the SARS-CoV-2 spike protein and firefly luciferase. After culturing in 293F cells (Thermo Fisher) for 3 days, viral supernatants were obtained. The neutralizing antibodies were serially diluted two-fold, starting from a 10 μg/mL concentration. The virus solution and antibody dilution were mixed in equal volumes and incubated for 1 hour at 37°C. Cell plates, pre-coated with poly-L-lysine (Sigma-Aldrich), received the addition of 2×10^4^ 293T cells (ATCC) to each well. Following this, the virus-antibody mixture was introduced, and the plates were incubated at 37°C for 72h. After this period, the supernatant was transferred to a white plate, and an equal volume of substrate solution was added. After incubating in the dark for 2 minutes, we measured the fluorescence response of the cell wells using a Biotek Cytation I microplate reader.

### Structure simulation and cartesian_ddG for functional prediction of allelic genes

The original structures of antigens and antibodies can be obtained from the Protein Data Bank (PDB, https://www.rcsb.org/) (33). The structure of R1-32 (7YDI) and LY-CoV1404 (7MMO) were acquired. Primary docking using ZDOCK (3.0.2) was employed to generate the initial conformation of the antigen-antibody complex (34), followed by simulations of the complete conformations using Rosetta SnugDock software (https://www.rosettacommons.org/) (35). Meanwhile, the cartesian_ddG functionality of the Rosetta software was utilized to predict the impact of single-point mutations on the energy of the complex structure. ΔΔG is defined as the difference in free energy changes (ΔG) between two different states, commonly used to compare the impact of mutations on molecular stability or interactions. Two ΔΔG values were predicted, and by calculating ΔΔG_mut - ΔΔG_wt, the influence of single-point mutations on the stability of the complex structure could be determined (36). The original and simulated structural conformations were visualized and presented using ChimeraX (v1.5) (37).

## Results

### IgH sequencing reveals IgH allelic gene polymorphism in the population

**Figure 1:**
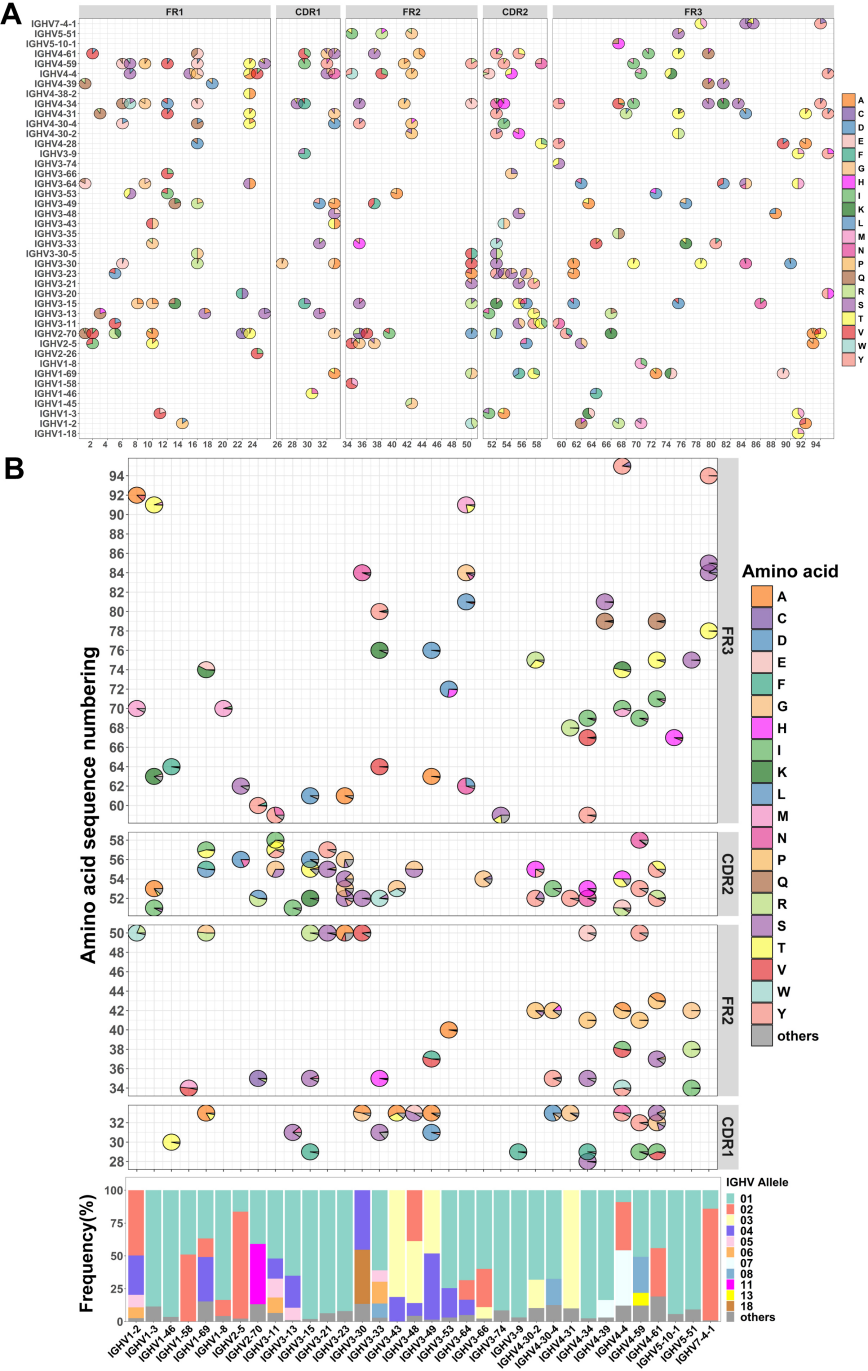
Investigation of allelic variants in the heavy chain variable region from 130 individuals. **(A)** Allelic variant maps of germline sequence in the IGHV. The bubble chart depicts the amino acid usage and proportions at each allele locus within each antibody family. **(B)** Allelic variant maps of the IGHV from 130 individuals. The frequency of allelic usage at each allele locus with each antibody family is shown in the sequence (top). Non-allelic variant amino acids are collectively categorized as “others” and represented in dark gray. The average frequency of allelic genotypes in the IGHV across 130 individuals (bottom). Allelic genotypes below 8% are uniformly categorized as “others” and indicated in dark gray.

Despite the increasing number of reports highlighting the potential impact of IgH allelic gene polymorphism on antibody function, a comprehensive analysis of IgH alleles is still lacking. To gain a more comprehensive understanding of the allelic genes of immunoglobulin heavy chain variable regions (IGHV), we first retrieved the germline sequences for the IGHV from the IMGT database (https://www.imgt.org/). We selected 42 functional IGHV families from 111 IGHV families to establish an allelic atlas (48 pseudogene families,16 lacking allele families and 5 unknown functional families were excluded) (**Figure 1A**; **Supplementary Figure S1A**). We identified 26 IGHV families with at least 5 distinct genotypes, including IGHV1-69 (20 genotypes), IGHV2-70 (19 genotypes), IGHV3-30 (20 genotypes), IGHV4-34 (13 genotypes), and IGHV4-59 (13 genotypes) (**Supplementary Figure S1B**, **Supplementary Table S1**). Subsequently, we detected a total of 238 allelic gene loci within the IGHV region, with 62 loci located in the FR1 region, 36 loci in the FR2 region, 78 loci in the FR3 region, 29 loci in the CDR1 region, and 33 loci in the CDR2 region (**Supplementary Figure S1C**, **Supplementary Table S1**). Notably, among these families were those exhibiting a high density of allele gene loci in the CDR1 and CDR2 regions such as IGHV1-69 (3 loci), IGHV2-5 (3 loci), IGHV3-15 (4 loci), IGHV3-23 (4 loci), IGHV3-30 (3 loci), IGHV4-34 (4 loci), and IGHV4-4 (4 loci) (**Supplementary Figure S1D**). Amino acid diversity in the CDR1 and CDR2 regions is related to binding affinity and neutralizing activity, as reported in several previous studies (38–40). Therefore, the large number of allelic loci within these regions suggests potential functional diversity. Overall, this comprehensive allelic atlas provides insight into the amino acid polymorphism present within the germline sequences of IGHV genes and illustrates the genotypic diversity of antibody response.

Although there have been some studies of immunoglobulin heavy chain alleles, few reports have focused on allelic distribution in populations. This distribution may be related to population immunity. To investigate the genetic variation in the population, we compiled the sequence data of the IgM and IgD repertoires of 130 individuals (21–23), including both healthy individuals (n=24) and COVID-19 convalescents (n=106). By tracking the antibody repertoire of six COVID-19 patients over the course of a year, we found that the IgM and IgD antibody repertoires during the infection period and one year later showed high similarity (**Supplementary Figures S4A, B**). A Wilcoxon test comparing allele usage in the IgM and IgD antibody repertoires during the infection period and one year later yielded a P-value of 0.8787, indicating that pathogenic infections have a limited impact on the IgM and IgD repertoires in individuals. Therefore, we used MiXCR and the IgM and IgD repertoire data to infer personal genotype by performing germline genotyping on the IgM and IgD sequences (28). After quality control processing of the sequencing data (19), we obtained 140 million valid IgH sequences. Of these, 29 million sequences correspond to the IgM and IgD repertoires. Excluding less prevalent IGHV families, we performed a statistical analysis of population allelic genotypes for 33 IGHV families. Despite the high diversity of allelic genotypes among different IGHV families (**Supplementary Figure S1B**), a few genotypes are prevalent in the population, including IGHV1-69*01 (36.7%), IGHV1-69*02 (14.1%), IGHV1-69*04 (33. 9%), IGHV2-5*02 (81.4%), IGHV2-70*01 (40.9%), IGHV2-70*11 (45.8%), IGHV3-30*04 (45.3%), IGHV3-30*18 (41.2%), IGHV3-53*01 (74.8%), IGHV4-34*01 (97.5%), IGHV4-59*01 (50.7%) and IGHV4-59*08 (27.4%) (**Figure 1B**). The above observation showed that most individuals have similar allelic genotypes.

### Certain allelic genotypes predominate in SARS-CoV-2 spike-specific antibodies

**Figure 2:**
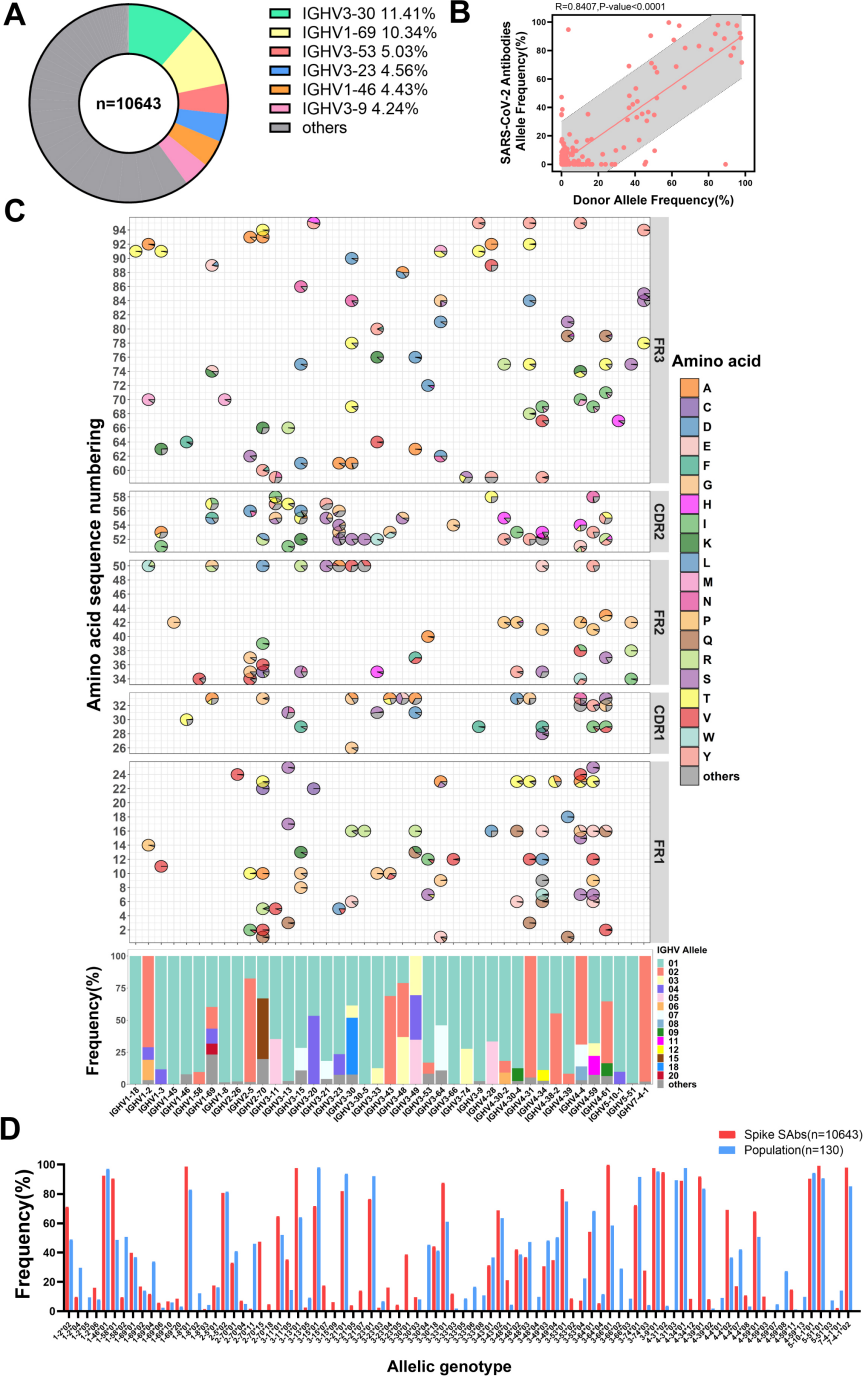
Specific antibodies utilize antibody allelic genotypes similar to those found in the population. **(A)** Doughnut chart displaying the antibody family usage frequencies among 10643 spike-specific antibodies. The top six most frequently used families are color-coded. **(B)** The allele frequencies correlation between SARS-CoV-2 specific antibodies and population. **(C)** Distribution of IGHV allelic usage in SARS-CoV-2 specific antibodies. The frequency of amino acid usage at each allele locus within each antibody family is shown in the FR1 to FR3 regions of IGHV (top). The horizontal axis corresponds to the antibody families. Non-allelic amino acids are grouped as “others” and represented in dark gray. The average frequency of allelic genotypes in the IGHV, with genotypes below 8% grouped as “others” and shown in dark gray (bottom). **(D)** Comparison of allelic genotypes usage frequencies between the population and specific antibodies. The frequency of allelic genotypes of more than 4% in groups is displayed.

To investigate the impact of SARS-CoV-2 infection on allelic gene usage in population-derived antibodies, we compiled a dataset of 10,643 SARS-CoV-2 spike-specific antibodies obtained from CoV-AbDab (29). We observed that IGHV3-30 antibodies (11.4%) were the most abundant, along with five other antibody families: IGHV1-69 (10.3%), IGHV3-53 (5.0%), IGHV3-23 (4.6%), IGHV1-46 (4.4%) and IGHV3-9 (4.2%), in agreement with our previous findings (35). Together, these families accounted for 39.6% of the total number of antibodies, while the remaining 45 IGHV families accounted for 60.1% (**Figure 2A**). We categorized these antibodies according to their allelic V genotype using the germline sequences provided by IMGT (28, 36). There is a strong correlation between the use of allelic genotypes found in the population and those present in spike-specific antibodies (**Figure 2B**). Furthermore, we presented the top 25 IGHV families in spike-specific antibodies and compared their allelic gene usage between individuals and specific antibodies pairwise. In general, alleles commonly observed in the population are also prevalent in SARS-CoV-2 spike-specific antibodies (**Figures 1B**, **2C**). However, the usage of several allelic genotypes is enriched in spike-specific antibodies. For example, IGHV1-2*02 is 21.1% more common in these antibodies than in the general population (71.0% vs. 49.9%). IGHV1-58*01 is 41.9% more frequent (90.4% vs. 48.5%), and IGHV4-4*02 is 28.6% more frequent (65.3% vs. 36.7%) (**Figure 2D**). These results suggest that certain allelic genotypes may enhance the affinity of antibodies to the SARS-CoV-2 spike protein.

### SARS-CoV-2 neutralizing antibodies with different allelic genes exhibit epitope-binding preferences

**Figure 3:**
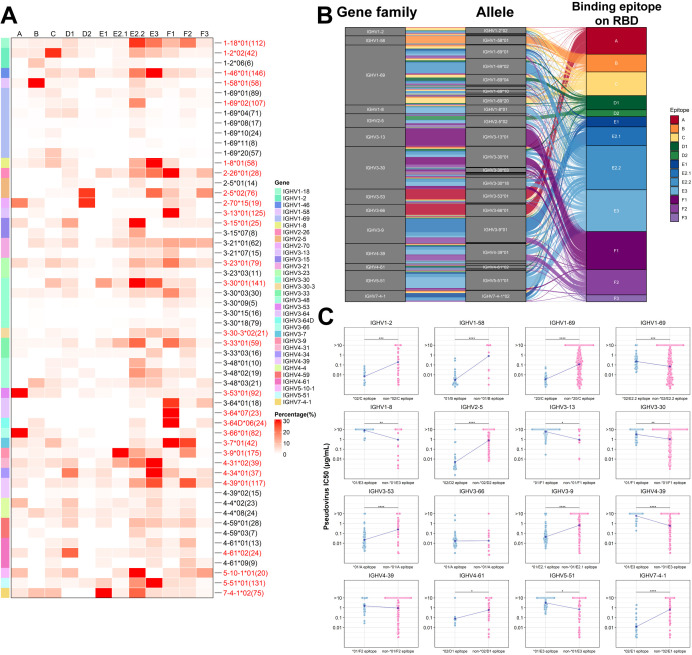
Preference of binding epitopes exhibited by different allelic variants of SARS-CoV-2 neutralizing antibodies. **(A)** The heatmap illustrates the association between allelic variants of neutralizing antibodies and binding epitopes. The number of antibodies corresponding to each allelic genotypes are labeled on the right. Allelic clusters with a high percentage are labeled in red font on the right side of the heatmap. **(B)** Sankey diagram demonstrates the enrichment of specific allelic clusters at distinct binding epitopes. Each line represents an antibody, and the color of the line indicates the binding epitope on the RBD. **(C)** Comparison of neutralization differences among different antibody clusters. Wilcoxon test was employed for comparisons. (*p<0.05, **p<0.01, ***p<0.001, ****p<0.0001).

We selected antibodies with neutralization data available from the CoV-AbDab database to assess the association between allelic genotypes and neutralizing epitopes (A-F3) on the RBD (31, 32). Based on these data, we combined the allelic genotypes with the binding epitopes, attempting to define different antibody clusters using the allelic genotype/epitope combinations. To simplify the nomenclatures, we abbreviate antibody clusters such as IGHV1-69*02 that bind to the E2.2 epitope as IGHV1-69*02 (E2.2 epitope), IGHV2-5*02 that bind to the D2 epitope as IGHV2-5*02(D2 epitope) and so on. To refine our analysis and avoid potential biases from small sample sizes, we filtered out the dataset with fewer than four antibodies per allelic genotype/epitope cluster. As a result, we identified 56 antibody clusters in 32 antibody families. We then identified the most prevalent antibody cluster within each antibody family and marked the corresponding 28 allelic genotypes (**Figure 3A**). 14 antibody families were selected for subsequent analysis based on the number of allelic sites and total antibody counts (**Figure 3B**). This analysis demonstrated that specific allelic genotypes within each family favor binding to distinct epitopes. In addition, a comparative evaluation of big antibody clusters with other antibodies from the same families revealed significant variances in neutralizing activity against the D614G strain among 14 antibody families, including IGHV1-2*02 (C epitope), IGHV1-69*02 (E2.2 epitope), IGHV1-69*20 (C epitope), IGHV2-5*02 (D2 epitope), IGHV3-53*01 (A epitope), IGHV5-51*01 (E3 epitope) (**Figure 3C**). Antibodies from IGHV1-69, using diverse allelic genotypes at specific epitopes, exhibited marked differences in neutralizing activity against D614G pseudoviruses. This observation suggests that different allelic genotypes contribute significantly to functionality at distinct epitopes, even within the same family. Previous research focused on the HCDR3 region’s primary role in determining antibody binding epitopes, with contributions from other regions of both the light and heavy chains (41–43). Our analysis revealed that different IgH allelic genotypes that exhibit epitope-binding preferences can also significantly contribute to different neutralizing potency and breadth. We subsequently carried out experiments to validate this finding using IGHV1-69 and IGHV2-5 antibodies against SARS-CoV-2.

### IGHV1-69*02 (E2.2 epitope) antibodies tend to have broad-spectrum neutralizing activities, while IGHV1-69*20 (C epitope) antibodies tend to have greater neutralizing potency

**Figure 4:**
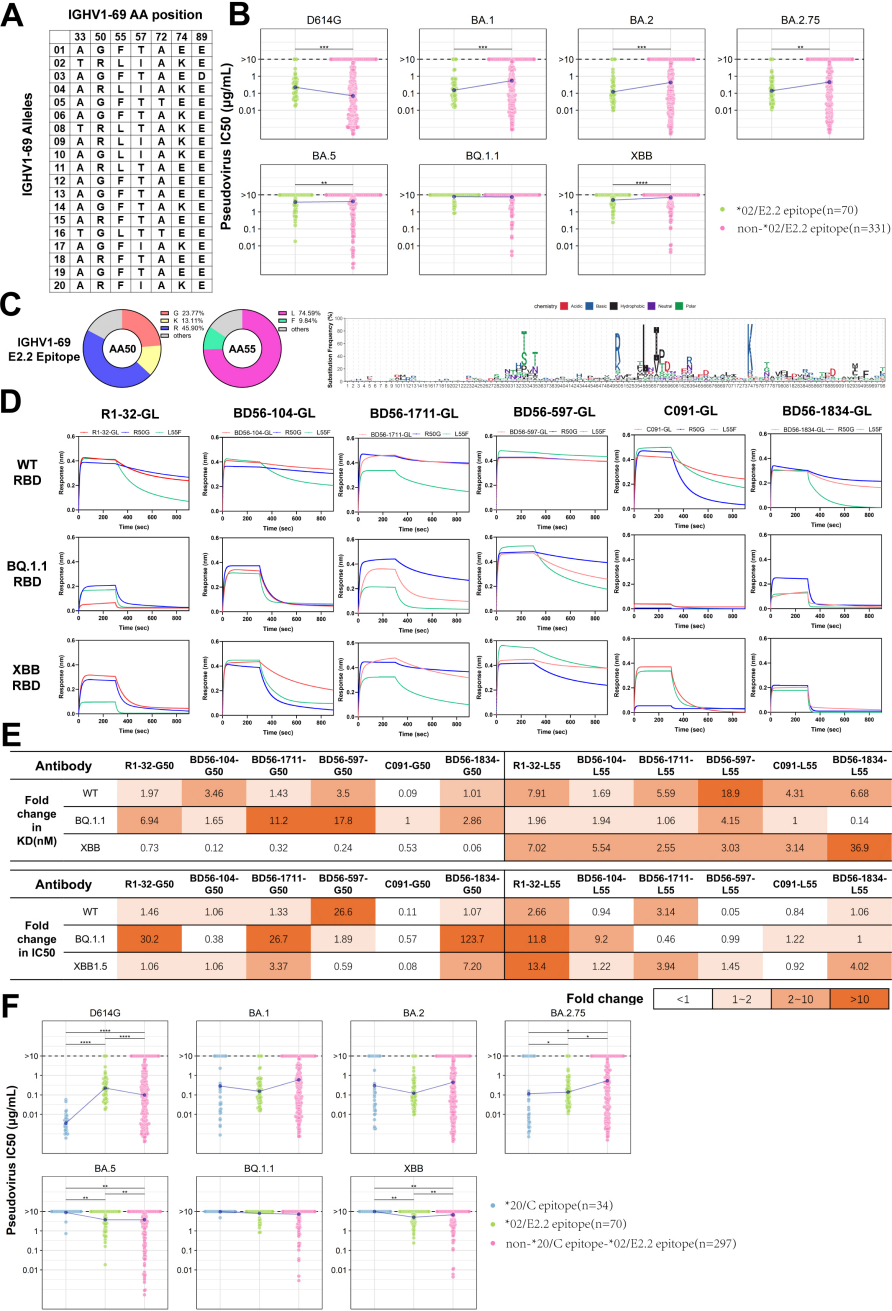
Analysis and Functional Validation of IGHV1-69 SARS-CoV-2 Neutralizing Antibodies **(A)** Amino acid map summarizes the positions of IGHV1-69 alleles. **(B)** Comparison of neutralizing activity between IGHV1-69*02(E2.2 epitope) and other IGHV1-69 nAbs against six SARS-CoV-2 variants. **(C)** Amino acid usage distribution maps for IGHV1-69(E2.2 epitope) nAbs. The doughnut charts display the frequency of amino acids at positions 50 and 55. The logo plot illustrates the amino acid usage in IGHV1-69(E2.2 epitope) antibodies. **(D)** Binding affinity comparison of six IGHV1-69/E2.2 antibodies to RBD from three SARS-CoV-2 strains. GL denotes antibodies with variable regions restored to germline sequences. **(E)** Comparison of binding affinity and neutralization fold change between two allelic variants against three mutant strains. The fold change of G50 and L55 is calculated as the ratio of R50 to G50, and the ratio of F55 to L55, respectively. **(F)** Comparison of neutralization among IGHV1-69*20(C epitope), IGHV1-69*02(E2.2 epitope), and other IGHV1-69 neutralizing antibodies against six SARS-CoV-2 variants. The Wilcoxon test was performed in **(B)** and **(F)** (*p<0.05, **p<0.01, ***p<0.001, ****p<0.0001).

The IGHV1-69 antibody family is characterized by multiple allelic gene sites within the VH gene (**Figure 4A**) and plays a crucial role in immune defense against SARS-CoV-2. We evaluated the neutralization potency of IGHV1-69*02 (E2.2 epitope) and other IGHV1-69 neutralizing antibodies against six Omicron variants (BA.1, BA.2, BA.2.75, BA.5, BQ.1.1 and XBB). According to the standards defined in the relevant literature, an IC50 value higher than 10 µg/mL is considered to have no neutralization (44). IGHV1-69*02 (E2.2 epitope) antibodies demonstrated broad-spectrum neutralizing activities across multiple variants, except BQ.1.1 (**Figure 4B**). Our findings revealed that approximately 60% of E2.2 epitope-binding antibodies utilize the basic amino acids, including the allelic variant R50 (45.90%) and the mutant K50 (13.11%), while 23.77% utilize the amino acid G50. The remaining 17.22% are composed of 9 different amino acids due to somatic hypermutation. Approximately 74.59% of E2.2 epitope-binding antibodies utilize the amino acid L55, while 9.84% utilize F55. The remaining 15.57% comprise 6 different amino acids owing to somatic hypermutation (**Figure 4C**).

To further analyze the IGHV1-69*02 (E2.2 epitope) antibodies, we employed R1-32, an IGHV1-69*10 (E2.2 epitope) antibody we identified in our lab. We have resolved the structural biology of the interaction between antibodies and RBD using cryo-EM as a model (40). R1-32 utilizes G50 and L55 in the IGHV region, as well as IGLV1-40, and shares a similar HCDR3 region, which is 17 amino acids long and contains the ‘GYSGYG’ or ‘GYSGSG’ motif. These characteristics are common among most IGHV1-69*02 (E2.2 epitope) antibodies. R1-32 is escaped by L452R mutation on RBD. Based on the established structure, we conducted simulations to evaluate the influence of the R50 and F55 on antigen-antibody interactions for both the wild-type L452 RBD and the R452 RBD (**Supplementary Figure S2A**). The results of the energy calculations indicated that R50 enhanced binding, whereas F55 weakened the interaction. The functional analysis was conducted using five R1-32-like antibodies, including R1-32, and one non-R1-32-like antibody, BD56-1834 (**Supplementary Figure S2B**). To circumvent the potential influence of somatic hypermutations (41, 42), all heavy chains were reverted to their IGHV1-69*02 germline sequences. The impact of these single-point mutations on antibody binding affinity was assessed through biolayer interferometry (BLI) experiments. In the case of the non-L452R RBD, both G50 and R50 demonstrated comparable binding affinities. However, when the binding of G50 and R50 to the L452R RBD of BQ.1.1 was tested, it was observed that G50 demonstrated greater binding resilience to L452R escape than R50 (**Figure 4D**). To understand the functional differences of the 55th position alleles, we changed these antibodies from L55 to F55 for comparison. The mean *k_on_* value of L55 antibodies is (3.75 ± 0.82) ×10^5^ M^−1^ s^−1^, while that of F55 antibodies is (3.50 ± 0.67) ×10^5^ M^−1^ s^−1^, demonstrating similar binding activities to the WT RBD. However, L55 antibodies showed a markedly slower dissociation rate (mean *k_off_* = (1.64 ± 0.50) ×10^-3^ s^−1^) than F55 antibodies (mean *k_off_* = (9.02 ± 3.80) ×10^-3^ s^−1^), significantly enhancing their binding affinity (**Figure 4E**). The difference in dissociation rates directly affected the affinity of L55(mean *K_D_* = 7.72 ± 4.52 nM) and F55(mean *K_D_* = 40.83 ± 25.62 nM) for WT RBD. Similar phenomena were also observed with the BQ.1.1 RBD and XBB RBD (**Supplementary Table S2**).

To investigate the role of allelic variants in neutralizing potency, we measured the half-maximal inhibitory concentration (IC_50_) against SARS-CoV-2 pseudoviruses. G50 variants exhibited comparable neutralizing activities to R50 against non-L452R strains, except for BD56-597. G50 variants demonstrated potential tolerance to the BQ.1.1 subvariant, which contains the L452R mutation (**Figure 4E**; **Supplementary Figure S2C**). Although the IGHV1-69 antibodies and their allelic variants showed significant differences in affinity, this didn’t fully translate to differences in neutralizing activity (**Supplementary Figures S2E, F**). Previous reports have suggested that a class of CAB-I47-like antibodies, using IGHV1-69*20 allelic genotypes, showed high affinity and neutralization activity against the SARS-CoV-2 WT strain (16). However, these antibodies, which also bound to the C epitope, were escaped by the Beta variants. We observed that IGHV1-69*20 (C epitope) antibodies exhibited enhanced neutralizing activities against the D614G variants but were more susceptible to escape by Omicron variants (**Figure 4F**). Notably, neutralizing antibodies binding to the C epitope predominantly employed allelic variants R50 and F55 rather than G50 and L55 (**Supplementary Figure S2D**). Consistent with our observation, CAB-I47-like antibodies belong to the IGHV1-69*20 (C epitope) clusters. These antibodies utilize R50 and F55, which exhibit enhanced binding affinity and neutralization. When different allelic variants, G50 or L55, were used, these antibodies exhibited a significantly reduced binding affinity and neutralization potency (16). Overall, antibodies binding the same epitope can exhibit varying binding affinity and neutralization potency against SARS-CoV-2 due to variations in their allelic genotypes.

### IGHV2-5*02 (D2 epitope) antibodies mostly exhibit broad-spectrum neutralizing activities

**Figure 5:**
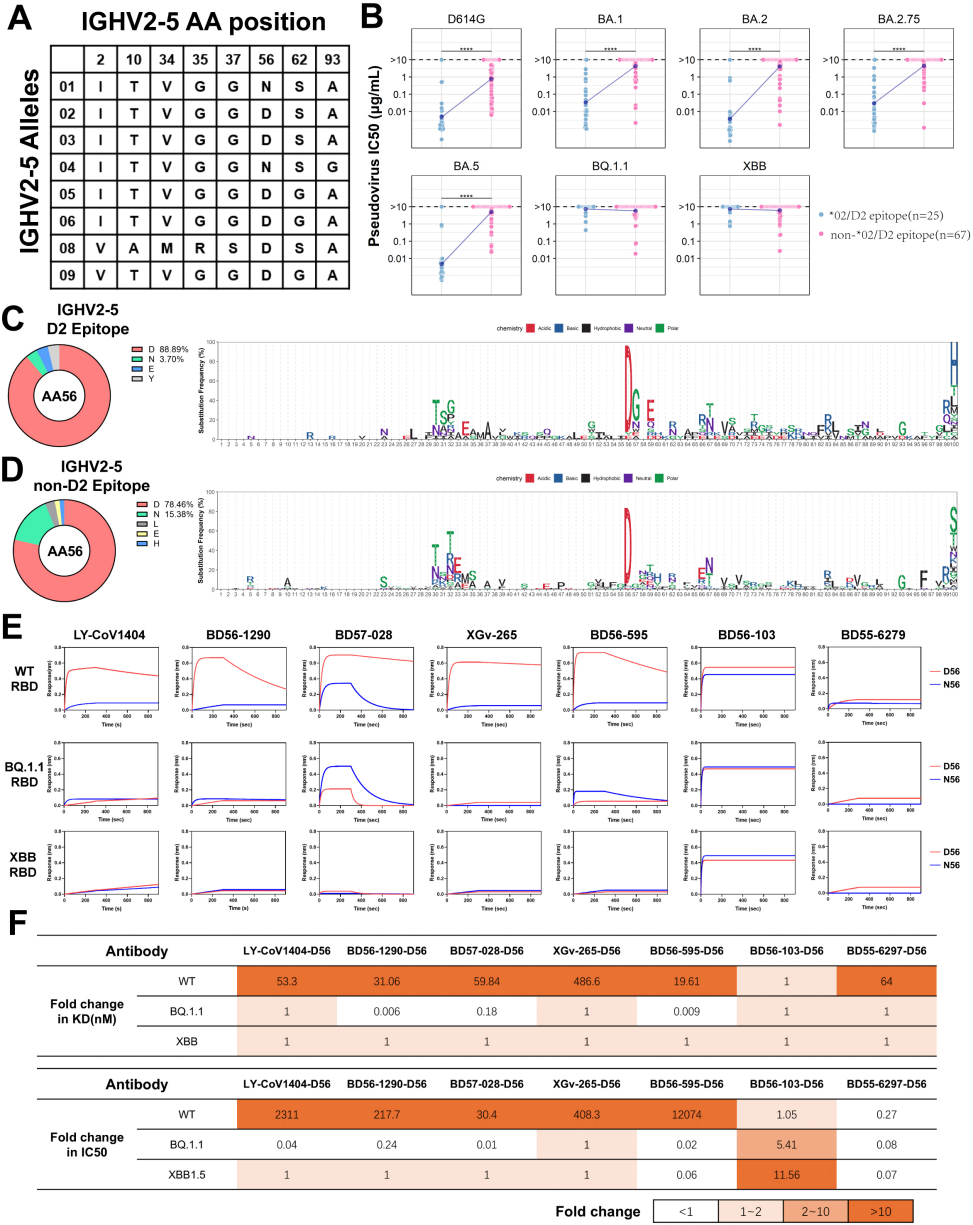
Analysis and Functional Validation of IGHV2-5 Neutralizing Antibodies **(A)** Amino acid profile of IGHV2-5 antibody allelic variant sites. **(B)** Comparison of neutralization by IGHV2-5*02(D2 epitope) and other IGHV2-5 neutralizing antibodies against different SARS-CoV-2 variants. Wilcoxon test was performed (****p<0.0001). **(C), (D)** Amino acid usage distribution of IGHV2-5*02(D2 epitope) and other IGHV2-5 antibodies. The logo plots show the amino acid usage in different IGHV2-5 antibody clusters. **(E)** Affinity comparison of five IGHV2-5*02(D2 epitope) antibodies and two non-D2 antibodies (F1/F3 epitope) and their allelic variants on 56th position with three different SARS-CoV-2 RBDs. **(F)** Comparison of binding affinity and neutralization fold change between D56 and N56 against three mutant strains. The fold change of D56 is calculated as the ratio of N56 to D56.

The IGHV2-5 antibodies have attracted attention for their prevalent occurrence in the antibody repertoire of SARS-CoV-2 vaccinees and their broad-spectrum neutralization (45–47). Analysis of the IGHV2-5 gene germline sequences from IMGT identified 8 allelic variant sites (**Figure 5A**). Unlike IGHV1-69, the allelic genotypes of IGHV2-5 in 130 individuals are predominantly concentrated in *01 and *02 (16.3% and 81.4%, respectively) in our study (**Figure 1B**), corresponding to the N/D difference at position 56 of CDR2. This data, derived from the IgM and IgD repertoires, is similar to that from an antibody repertoire analysis of 13 individuals, where the frequency of IGHV2-5*02 and IGHV2-5*01 accounted for 64% and 33% among all IGHV2-5 antibodies, respectively (15). In line with population allelic frequency data, analysis of IGHV2-5 spike-specific antibodies revealed a predominance of allelic genotypes IGHV2-5*02 (80.6%) (**Figure 2C**). We classified IGHV2-5 neutralizing antibodies against RBD based on allelic genotypes and epitopes. IGHV2-5*02 (D2 epitope) antibodies exhibit broad-spectrum neutralizing activities against D614G, BA.1, BA.2, BA.2.75, and BA.5 variants, until evasion by BQ.1.1 and XBB (**Figure 5B**). Interestingly, the amino acid residue at position 56 in IGHV2-5 antibodies varies according to the binding epitope. 88.9% of IGHV2-5 (D2 epitope) neutralizing antibodies utilize D56, with 3.7% utilizing N56 (**Figure 5C**). 78.46% of IGHV2-5 (non-D2 epitope) antibodies utilize N56, while 15.38% utilize D56 (**Figure 5D**), suggesting both D56 and N56 residues might contribute to neutralization positively. To investigate the functional aspects of IGHV2-5*02 (D2 epitope) antibody genotypes, we used LY-CoV1404 as a base and employed structural simulation to predict the functional roles of the genotypes. The simulation revealed that D56 could form hydrogen bonds and salt bridges with K444 on WT RBD protein, contributing to the formation of a stabilized structure, which was in line with previous reports (48). In contrast, N56 was unable to bind with K444. Upon introducing a K444T single-point mutation on the WT RBD, D56 failed to form a salt bridge with T444, resulting in a weakened force in the CDR2 region (**Supplementary Figure S3A**), consistent with previous reports (48, 49).

To clarify the relationship between alleles and epitopes, we selected five IGHV2-5*02 (D2 epitope) antibodies, LYCoV-1404, BD56-1290, BD57-028, XGv-265, BD56-595, and two non-D2 epitope antibodies, BD56-103 (IGHV2-5*01/F1 epitope) and BD55-6297 (IGHV2-5*01/F3 epitope) from the neutralizing antibodies database, for further analysis. These five antibodies predominantly shared IGHJ4 and are paired with IGLV2-14, with HCDR3 regions typically consisting of 11 amino acids (**Supplementary Figure S3B**).

IGHV2-5*02 (D2 epitope) neutralizing antibodies possessing the D56 allele exhibited high affinity for the WT RBD. In contrast, the N56 allele showed almost no binding to the WT-RBD, as demonstrated by BLI analysis (**Figure 5E**). Interestingly, IGHV2-5*02 (D2 epitope) antibodies, like BD57-028 and BD56-595, with the N56 allelic variant, showed a 57-fold increase in binding affinities to BQ.1.1-RBD carrying K444T mutation compared to those with the D56 variant (**Figure 5F**; **Supplementary Table S3**). In contrast, for IGHV2-5*01 (F1 epitope) and IGHV2-5*01 (F3 epitope) antibodies, there was no significant difference in the impact of D56 and N56 on binding affinity (**Figure 5E**). We analyzed the neutralizing activities of IGHV2-5 antibodies against the pseudoviruses WT, BQ.1.1, and XBB1.5 (**Supplementary Figure S3C**). D56 significantly enhanced the neutralizing activity of IGHV2-5*02(D2 epitope) antibodies against the WT pseudovirus compared to N56 (**Figure 5F**), which aligned with the BLI results (**Supplementary Figure S3D**). Interestingly, the allelic variant D56 of the IGHV2-5*01 (F1 epitope) antibody BD56-103 exhibited greater tolerance to mutation escape. In contrast, the N56 allele of the IGHV2-5*01(F3 epitope) antibody BD55-6297 significantly improved the antibody’s neutralizing activity against all three SARS-CoV-2 strains (**Supplementary Figures S3C, E**). These results suggest that the functionality of IgH allelic genes is closely associated with the antibody-binding epitopes. In other words, antibodies with the same allelic gene can exhibit distinct functions across different epitopes. Furthermore, antibodies with specific allelic genotypes and binding epitopes can show resilience to SARS-CoV-2 mutation escape.

## Discussion

With the recent global outbreak of the COVID-19 pandemic and the application of high-throughput sequencing technologies, it has become possible to study the immune mechanism from a molecular biology perspective (50, 51). There is a growing interest in the foundational research of population immunology. Ig alleles play a crucial role in shaping the humoral immune response, particularly in the context of antibody diversity and specificity. The ability of the human immune system to recognize and neutralize a wide array of pathogens is significantly influenced by the polymorphisms in Ig genes. Furthermore, Ig alleles can influence susceptibility to various diseases. For example, one study showed that the R110 allelic variant of IGLV3-32*01 is often associated with poor prognosis in chronic lymphocytic leukemia (CLL) (52). Despite this, there has been a lack of comprehensive research focusing on how specific Ig alleles affect the function and efficacy of antibodies, especially in response to emerging pathogens such as SARS-CoV-2. This study provided an allelic atlas of IgH variable regions, demonstrating their genetic diversity and functional relevance in the context of SARS-CoV-2 infection. Our analysis identified and characterized the diversity of allelic variants across 33 antibody family germline sequences through IgH repertoire sequencing of 130 individuals, encompassing both SARS-CoV-2 infected people and healthy donors. The comprehensive analysis of approximately 10,000 SARS-CoV-2 specific antibodies revealed that certain IgH alleles are preferentially utilized in the humoral immune response against SARS-CoV-2. These findings highlighted that genetic diversity within the population can influence the distribution and prevalence of specific immunoglobulin alleles. One study showed that the frequency of IGHV2-5*01 (N56) antibodies is 33% among all IGHV2-5 antibodies in the antibody repertoire of 13 healthy volunteers. But no IGHV2-5*01 was used among RBD-specific antibodies encoded by IGHV2-5/IGLV2-14 (15). Although it is unclear whether this process arises from selection or mutations from N to D in the alleles, it provides strong evidence that favorable residues can be enriched under certain circumstance. However, we did not observe a significant enrichment of G50 or R50 among IGHV1-69 antibodies in our longitudinal analysis of COVID-19 convalescents, which may be related to the functional diversity of the IGHV1-69 alleles. The diversity of alleles can affect the population’s binding resilience to SARS-CoV-2 variants. Insights gained from this research can inform public health strategies and personalized medicine approaches, ensuring that interventions are equitable and effective across diverse genetic backgrounds.

Our study also revealed epitope preference in the usage of alleles in the heavy chain of RBD-binding antibodies, meaning that certain alleles are more likely to recognize and bind to specific epitopes of SARS-CoV-2. This finding implies that allele preference may play an important role in antiviral immune responses, contributing to our understanding of the functional properties of antibodies and immune response mechanisms. Previous studies showed that CAB-I47 possessed strong neutralizing activity against WT strain owing to the use of R50 and F55 residues, while the use of G50 and L55 residues could completely abolish both binding and neutralizing activity (16). According to our findings, CAB-I47 belongs to IGHV1-69*20 (C epitope). All IGHV1-69*20 (C epitope) antibodies had significantly enhanced binding and neutralizing activity compared to antibodies from other clusters. Our study found no significant difference in the functionality of R50 and G50 residues in IGHV1-69*02 (E2.2 epitope) antibodies when interacting with the SARS-CoV-2 spike carrying the L452. However, the potential repulsion between R50 and the L452R mutation reduced the binding affinity of R50 for the RBD, making G50 more tolerant to the SARS-CoV-2 carrying spike with the L452R mutation. On the other hand, F55 and L55 exhibit similar binding kinetics to the RBD of SARS-CoV-2 different mutant strains. However, the faster dissociation rate of F55 suggests potential steric hindrance effects. These effects may occur in the hydrophobic environment of the interaction between the IGHV1-69*02 (E2.2 epitope) antibody F55 and spike with L452, suggesting L55 might be a more favorable choice. Recent studies have also shown that within a series of IGHV1-69 antibodies against H1N1, F55 plays a key role in binding the influenza HA stem. However, L55 binds and neutralizes a broader range of influenza HAs and is more suited for further affinity maturation (53), aligning closely with our findings. Interestingly, a number of IGHV1-69*02 (E2.2 epitope) neutralizing antibodies using R50 and F55 were identified, rather than IGHV1-69*10 (E2.2 epitope) or IGHV1-69*16 (E2.2 epitope) using G50 and L55. We speculate that this could be due to two factors. First, the IGHV1-69*02 allelic genotype is more common in the population. Second, the neutralizing antibodies in the database are predominantly from vaccine recipients or convalescents exposed to non-L452R mutant strains.

The IGHJ and IGLJ differ among selected IGHV1-69 and IGHV2-5 antibodies. However, our structural analysis showed that J genes did not interact with the spike protein, so we did not consider these differences in our study. Through structural simulations and single amino acid site energy calculations, A33T had less than 0.6 kcal/mol in both WT-RBD and L452R-RBD (0.585 and 0.254, respectively). We believe that A33 and T33 have a similar impact on antibodies. We also found that I57 (mean *K_D_* = 0.72±0.23 nM) and T57 (mean *K_D_* = 0.98±0.28 nM) have similar functions in antibodies against WT RBD based on BLI experiments. Therefore, our selected antibodies include IGHV1-69*02, IGHV1-69*08 and IGHV1-69*10 antibodies. BD56-1834, which is not an R1-32 like antibody, has similar function to R1-32 like antibodies. We speculate that this is mainly related to the binding epitope. The differences among the selected antibodies may be attributed to variations in the heavy chain CDR3 region and the light chain.

Previous studies defined the IGHV2-5*02 (D2 epitope) antibody, represented by LY-CoV1404, along with its D56 allelic variant. This antibody exerts strong forces through hydrogen bonds and salt bridges with K444 on spike RBD (48). However, with the emergence of the K444T mutation (49), the BQ.1.1 variant, as a representative, made IGHV2-5*01 (D2 epitope) with N56 a more favorable selection. Upon leaving the RBD D2 epitope, the roles of allelic genotypes IGHV2-5*01 and IGHV2-5*02 might change, with N56 (IGHV2-5*01) enhancing antibody neutralization against the WT, BQ.1.1, and XBB1.5 mutant strains more than D56 (IGHV2-5*02).

There are several limitations in this study. Firstly, the sample size of the cohort, consisting of 130 individuals, is relatively small and may not adequately represent a broader population. The spike-specific antibodies were sourced from public databases and screened from diverse populations worldwide. Therefore, the comparison between the population and spike-specific antibodies might lead to results that are not sufficiently rigorous. To address these issues, large-scale whole-genome sequencing and BCR repertoire sequencing of COVID-19 patients or convalescents are needed for future studies. Secondly, the spike-specific antibody sequences are derived from amino acid sequences only. Therefore, all sequence alignments are based on differences in amino acid sequences without considering factors such as somatic hypermutation, and thus may not accurately reflect the actual nucleic acid genotypes. IGHV allele annotations are based on the IMGT germline reference, which has limited genomic information and does not fully capture the genetic diversity of various populations (54). With advancements in sequencing technology and analytical tools, a large number of novel alleles are waiting to be discovered (55). Finally, this study attempted to elucidate the specific mechanisms of allelic variants through structural simulation, which may introduce some degree of uncertainty.

In summary, this study provides an allelic atlas of IgH variable regions and demonstrates the critical role of immunoglobulin alleles in shaping the humoral immune response to SARS-CoV-2. Our study demonstrated that the allelic genotypes and binding epitopes of antibodies can influence the function of antibodies, suggesting that the vaccine design could be tailored based on specific antigenic epitopes and the allelic genotypes of individuals. Such a strategy may improve the production of high-affinity and broad-spectrum antibodies, and therefore enhance vaccine efficacy.

## Data Availability

The original contributions presented in the study are publicly available. This data can be found here: PRJCA030431.

## Supplementary material

Supplementary Figure 1 Summary of IGHV germline sequence **(A)** Distribution of 111 IGHV families. **(B)** Comparison of the number of allelic genotypes across 42 antibody families. **(C)** Comparison of allelic site counts(aa) in each IGHV region. **(D)** The number of allelic gene loci in CDR1 and CDR2 regions of 26 antibody families.

Supplementary Figure 2 Sequence Information on IGHV1-69 Neutralizing Antibodies **(A)** Structural simulations contrasting the interactions of R1-32, R1-32-G50R, R1-32-L55F with WT RBD and L452R-RBD. Energy calculations are employed to predict the impact of different allelic variants. **(B)** Sequence information of IGHV1-69 monoclonal antibodies binding the E2.2 epitope. **(C)** The neutralization against WT, BQ.1.1, and XBB1.5 SARS-CoV-2 strains by IGHV1-69 monoclonal antibodies and their variants. **(D)** Amino acid usage distribution for IGHV1-69*20/C and remaining antibodies. **(E)** Correlation between binding affinity and neutralization of six IGHV1-69(E2.2 epitope) monoclonal antibodies. Dotted lines represent the detection limit. Best-fit lines were determined through simple linear regression analysis. Two-tailed Pearson correlation was employed to calculate the R and P values.

Supplementary Figure 3 Sequence Information of IGHV2-5 Neutralizing Antibodies **(A)** Structural comparison of the interaction between LY-CoV1404 and LY-CoV1404-D56N with WT RBD and K444T-RBD, respectively. Energy calculations are employed to predict the impact of different allelic variants. **(B)** Sequence information of five IGHV2-5*02(D2 epitope) and two non-D2 epitope monoclonal antibodies. **(C)** Neutralization against WT, BQ.1.1, and XBB1.5 SARS-CoV-2 strains by IGHV2-5 antibodies and their allelic variants. The red line represents D56 variants, while the blue line represents N56 variants. **(D)** Correlation between binding affinity and neutralization of seven IGHV2-5 monoclonal antibodies. Dotted lines represent the detection limit. Best-fit lines were determined through simple linear regression analysis. Two-tailed Pearson correlation was employed to calculate the R and P values.

Supplementary Figure 4 Comparison of IgM and IgD repertoires during infection and one year post-infection **(A)** Genotype frequency distribution of IgM and IgD repertoires in COVID-19 infected individuals. **(B)** Genotype frequency distribution of IgM and IgD repertoires in COVID-19 convalescents. Allelic genotypes with frequencies below 8% were categorized as “others”. Wilcoxon test was employed for comparisons.

## References

[1] Knoll MD Wonodi C. Oxford-AstraZeneca COVID-19 vaccine efficacy. Lancet. (2021) 397:72–4. doi: 10.1016/S0140-6736(20)32623-4 PMC7832220 33306990

[2] Polack FP Thomas SJ Kitchin N Absalon J Gurtman A Lockhart S. Safety and efficacy of the BNT162b2 mRNA covid-19 vaccine. N Engl J Med. (2020) ed2020:2603–15. doi: 10.1056/NEJMoa2034577 PMC7745181 33301246

[3] Tregoning JS Flight KE Higham SL Wang Z Pierce BF. Progress of the COVID-19 vaccine effort: viruses, vaccines and variants versus efficacy, effectiveness and escape. Nat Rev Immunol. (2021) 21:626–36. doi: 10.1038/s41577-021-00592-1 PMC8351583 34373623

[4] Chi WY Li YD Huang HC Chan TEH Chow SY Su JH. COVID-19 vaccine update: vaccine effectiveness, SARS-CoV-2 variants, boosters, adverse effects, and immune correlates of protection. J BioMed Sci. (2022) 29:82. doi: 10.1186/s12929-022-00853-8 36243868 PMC9569411

[5] Ebinger JE Fert-Bober J Printsev I Wu M Sun N Prostko JC. Antibody responses to the BNT162b2 mRNA vaccine in individuals previously infected with SARS-CoV-2. Nat Med. (2021) 27:981–4. doi: 10.1038/s41591-021-01325-6 PMC8205849 33795870

[6] Knies A Ladage D Braun RJ Kimpel J Schneider M. Persistence of humoral response upon SARS-CoV-2 infection. Rev Med Virol. (2022) 32:e2272. doi: 10.1002/rmv.v32.2 34191369 PMC8420449

[7] Zuo F Abolhassani H Du L Piralla A Bertoglio F de Campos-Mata L. Heterologous immunization with inactivated vaccine followed by mRNA-booster elicits strong immunity against SARS-CoV-2 Omicron variant. Nat Commun. (2022) 13:2670. doi: 10.1038/s41467-022-30340-5 35562366 PMC9106736

[8] Rodriguez OL Safonova Y Silver CA Shields K Gibson WS Kos JT. Genetic variation in the immunoglobulin heavy chain locus shapes the human antibody repertoire. Nat Commun. (2023) 14:4419. doi: 10.1038/s41467-023-40070-x 37479682 PMC10362067

[9] Watson CT Glanville J Marasco WA. The individual and population genetics of antibody immunity. Trends Immunol. (2017) 38:459–70. doi: 10.1016/j.it.2017.04.003 PMC5656258 28539189

[10] Chi X Li Y Qiu X. V(D)J recombination, somatic hypermutation and class switch recombination of immunoglobulins: mechanism and regulation. Immunology. (2020) 160:233–47. doi: 10.1111/imm.v160.3 PMC7341547 32031242

[11] Warrender AK Kelton W. Beyond allotypes: the influence of allelic diversity in antibody constant domains. Front Immunol. (2020) 11:2016. doi: 10.3389/fimmu.2020.02016 32973808 PMC7461860

[12] Avnir Y Watson CT Glanville J Peterson EC Tallarico AS Bennett AS. IGHV1-69 polymorphism modulates anti-influenza antibody repertoires, correlates with IGHV utilization shifts and varies by ethnicity. Sci Rep. (2016) 6:20842. doi: 10.1038/srep20842 26880249 PMC4754645

[13] Pappas L Foglierini M Piccoli L Kallewaard NL Turrini F Silacci C. Rapid development of broadly influenza neutralizing antibodies through redundant mutations. Nature. (2014) 516:418–22. doi: 10.1038/nature13764 25296253

[14] Ying T Prabakaran P Du L Shi W Feng Y Wang Y. Junctional and allele-specific residues are critical for MERS-CoV neutralization by an exceptionally potent germline-like antibody. Nat Commun. (2015) 6:8223. doi: 10.1038/ncomms9223 26370782 PMC4571279

[15] Yuan M Wang Y Lv H Tan TJC Wilson IA Wu NC. Molecular analysis of a public cross-neutralizing antibody response to SARS-CoV-2. Cell Rep. (2022) 41:111650. doi: 10.1016/j.celrep.2022.111650 36335937 PMC9606039

[16] Pushparaj P Nicoletto A Sheward DJ Das H Castro Dopico X Perez Vidakovics L. Immunoglobulin germline gene polymorphisms influence the function of SARS-CoV-2 neutralizing antibodies. Immunity. (2023) 56:193–206 e7. doi: 10.1016/j.immuni.2022.12.005 36574772 PMC9742198

[17] Sangesland M Torrents de la Pena A Boyoglu-Barnum S Ronsard L Mohamed FAN Moreno TB. Allelic polymorphism controls autoreactivity and vaccine elicitation of human broadly neutralizing antibodies against influenza virus. Immunity. (2022) 55:1693–709 e8. doi: 10.1016/j.immuni.2022.07.006 35952670 PMC9474600

[18] Dunbar J Krawczyk K Leem J Baker T Fuchs A Georges G. SAbDab: the structural antibody database. Nucleic Acids Res. (2014) 42:D1140–6. doi: 10.1093/nar/gkt1043 PMC3965125 24214988

[19] Yuan M Feng Z Lv H So N Shen IR Tan TJC. Widespread impact of immunoglobulin V-gene allelic polymorphisms on antibody reactivity. Cell Rep. (2023) 42:113194. doi: 10.1016/j.celrep.2023.113194 37777966 PMC10636607

[20] Ohlin M Scheepers C Corcoran M Lees WD Busse CE Bagnara D. Inferred allelic variants of immunoglobulin receptor genes: A system for their evaluation, documentation, and naming. Front Immunol. (2019) 10:435. doi: 10.3389/fimmu.2019.00435 30936866 PMC6431624

[21] Yan Q Zhang Y Hou R Pan W Liang H Gao X. Deep immunoglobulin repertoire sequencing depicts a comprehensive atlas of spike-specific antibody lineages shared among COVID-19 convalescents. Emerg Microbes Infect. (2023) 13:2290841. doi: 10.1080/22221751.2023.2290841 PMC10810631 38044868

[22] Zhang Y Yan Q Luo K He P Hou R Zhao X. Analysis of B cell receptor repertoires reveals key signatures of the systemic B cell response after SARS-coV-2 infection. J Virol. (2022) 96:e0160021. doi: 10.1128/jvi.01600-21 34878902 PMC8865482

[23] Niu X Li S Li P Pan W Wang Q Feng Y. Longitudinal analysis of T and B cell receptor repertoire transcripts reveal dynamic immune response in COVID-19 patients. Front Immunol. (2020) 11:582010. doi: 10.3389/fimmu.2020.582010 33117392 PMC7561365

[24] Wendel BS He C Qu M Wu D Hernandez SM Ma KY. Accurate immune repertoire sequencing reveals malaria infection driven antibody lineage diversification in young children. Nat Commun. (2017) 8:531. doi: 10.1038/s41467-017-00645-x 28912592 PMC5599618

[25] Bashford-Rogers RJM Bergamaschi L McKinney EF Pombal DC Mescia F Lee JC. Analysis of the B cell receptor repertoire in six immune-mediated diseases. Nature. (2019) 574:122–6. doi: 10.1038/s41586-019-1595-3 PMC6795535 31554970

[26] Bolger AM Lohse M Usadel B. Trimmomatic: a flexible trimmer for Illumina sequence data. Bioinformatics. (2014) 30:2114–20. doi: 10.1093/bioinformatics/btu170 PMC4103590 24695404

[27] Magoc T Salzberg SL. FLASH: fast length adjustment of short reads to improve genome assemblies. Bioinformatics. (2011) 27:2957–63. doi: 10.1093/bioinformatics/btr507 PMC3198573 21903629

[28] Bolotin DA Poslavsky S Mitrophanov I Shugay M Mamedov IZ Putintseva EV. MiXCR: software for comprehensive adaptive immunity profiling. Nat Methods. (2015) 12:380–1. doi: 10.1038/nmeth.3364 25924071

[29] Raybould MIJ Kovaltsuk A Marks C Deane CM. CoV-AbDab: the coronavirus antibody database. Bioinformatics. (2021) 37:734–5. doi: 10.1093/bioinformatics/btaa739 PMC7558925 32805021

[30] Ye J Ma N Madden TL Ostell JM. IgBLAST: an immunoglobulin variable domain sequence analysis tool. Nucleic Acids Res. (2013) 41:W34–40. doi: 10.1093/nar/gkt382 PMC3692102 23671333

[31] Cao Y Wang J Jian F Xiao T Song W Yisimayi A. Omicron escapes the majority of existing SARS-CoV-2 neutralizing antibodies. Nature. (2022) 602:657–63. doi: 10.1038/s41586-021-04385-3 PMC8866119 35016194

[32] Cao Y Yisimayi A Jian F Song W Xiao T Wang L. BA.2.12.1, BA.4 and BA.5 escape antibodies elicited by Omicron infection. Nature. (2022) 608:593–602. doi: 10.1038/s41586-022-04980-y 35714668 PMC9385493

[33] Berman HM Westbrook J Feng Z Gilliland G Bhat TN Weissig H. The protein data bank. Nucleic Acids Res. (2000) 28:235–42. doi: 10.1093/nar/28.1.235 PMC102472 10592235

[34] Pierce BG Wiehe K Hwang H Kim BH Vreven T Weng Z. ZDOCK server: interactive docking prediction of protein-protein complexes and symmetric multimers. Bioinformatics. (2014) 30:1771–3. doi: 10.1093/bioinformatics/btu097 PMC4058926 24532726

[35] Weitzner BD Jeliazkov JR Lyskov S Marze N Kuroda D Frick R. Modeling and docking of antibody structures with Rosetta. Nat Protoc. (2017) 12:401–16. doi: 10.1038/nprot.2016.180 PMC5739521 28125104

[36] Valanciute A Nygaard L Zschach H Maglegaard Jepsen M Lindorff-Larsen K Stein A. Accurate protein stability predictions from homology models. Comput Struct Biotechnol J. (2023) 21:66–73. doi: 10.1016/j.csbj.2022.11.048 36514339 PMC9729920

[37] Pettersen EF Goddard TD Huang CC Meng EC Couch GS Croll TI. UCSF ChimeraX: Structure visualization for researchers, educators, and developers. Protein Sci. (2021) 30:70–82. doi: 10.1002/pro.v30.1 32881101 PMC7737788

[38] Tian X Zhu X Song W Yang Z Wu Y Ying T. The prominent role of a CDR1 somatic hypermutation for convergent IGHV3-53/3-66 antibodies in binding to SARS-CoV-2. Emerg Microbes Infect. (2022) 11:1186–90. doi: 10.1080/22221751.2022.2063074 PMC9045774 35380101

[39] Yu H Liu B Zhang Y Gao X Wang Q Xiang H. Somatically hypermutated antibodies isolated from SARS-CoV-2 Delta infected patients cross-neutralize heterologous variants. Nat Commun. (2023) 14:1058. doi: 10.1038/s41467-023-36761-0 36828833 PMC9951844

[40] Chen W Li W Ying T Wang Y Feng Y Dimitrov DS. Germlining of the HIV-1 broadly neutralizing antibody domain m36. Antiviral Res. (2015) 116:62–6. doi: 10.1016/j.antiviral.2015.02.001 PMC4357557 25676867

[41] Chiu ML Goulet DR Teplyakov A Gilliland GL. Antibody structure and function: the basis for engineering therapeutics. Antibodies (Basel). (2019) 8:55. doi: 10.3390/antib8040055 31816964 PMC6963682

[42] Jaffe DB Shahi P Adams BA Chrisman AM Finnegan PM Raman N. Functional antibodies exhibit light chain coherence. Nature. (2022) 611:352–7. doi: 10.1038/s41586-022-05371-z PMC9607724 36289331

[43] Xu JL Davis MM. Diversity in the CDR3 region of V(H) is sufficient for most antibody specificities. Immunity. (2000) 13:37–45. doi: 10.1016/S1074-7613(00)00006-6 10933393

[44] Cao Y Jian F Zhang Z Yisimayi A Hao X Bao L. Rational identification of potent and broad sarbecovirus-neutralizing antibody cocktails from SARS convalescents. Cell Rep. (2022) 41:111845. doi: 10.1016/j.celrep.2022.111845 36493787 PMC9712074

[45] Guo Y Zhang G Yang Q Xie X Lu Y Cheng X. Discovery and characterization of potent pan-variant SARS-CoV-2 neutralizing antibodies from individuals with Omicron breakthrough infection. Nat Commun. (2023) 14:3537. doi: 10.1038/s41467-023-39267-x 37322000 PMC10267556

[46] Andreano E Paciello I Piccini G Manganaro N Pileri P Hyseni I. Hybrid immunity improves B cells and antibodies against SARS-CoV-2 variants. Nature. (2021) 600:530–5. doi: 10.1038/s41586-021-04117-7 PMC8674140 34670266

[47] Andreano E Paciello I Marchese S Donnici L Pierleoni G Piccini G. Anatomy of Omicron BA.1 and BA.2 neutralizing antibodies in COVID-19 mRNA vaccinees. Nat Commun. (2022) 13:3375. doi: 10.1038/s41467-022-31115-8 35697673 PMC9189263

[48] Westendorf K Zentelis S Wang L Foster D Vaillancourt P Wiggin M. LY-CoV1404 (bebtelovimab) potently neutralizes SARS-CoV-2 variants. Cell Rep. (2022) 39:110812. doi: 10.1016/j.celrep.2022.110812 35568025 PMC9035363

[49] Wang X Yang Y Song Z Wang Y Yang P Li X. Concerns on Bebtelovimab (LY-CoV1404) used to neutralize Omicron subvariants. J Med Virol. (2023) 95:e28565. doi: 10.1002/jmv.28565 36756927

[50] Huang Q Han X Yan J. Structure-based neutralizing mechanisms for SARS-CoV-2 antibodies. Emerg Microbes Infect. (2022) 11:2412–22. doi: 10.1080/22221751.2022.2125348 PMC9553185 36106670

[51] Wang H Liu C Xie X Niu M Wang Y Cheng X. Multi-omics blood atlas reveals unique features of immune and platelet responses to SARS-CoV-2 Omicron breakthrough infection. Immunity. (2023) 56:1410–28 e8. doi: 10.1016/j.immuni.2023.05.007 37257450 PMC10186977

[52] Maity PC Bilal M Koning MT Young M van Bergen CAM Renna V. IGLV3-21*01 is an inherited risk factor for CLL through the acquisition of a single-point mutation enabling autonomous BCR signaling. Proc Natl Acad Sci U S A. (2020) 117:4320–7. doi: 10.1073/pnas.1913810117 PMC7049113 32047037

[53] McIntire KM Meng H Lin TH Kim W Moore NE Han J. Maturation of germinal center B cells after influenza virus vaccination in humans. J Exp Med. (2024) 221:e20240668. doi: 10.1084/jem.20240668 38935072 PMC11211068

[54] Sherman RM Salzberg SL. Pan-genomics in the human genome era. Nat Rev Genet. (2020) 21:243–54. doi: 10.1038/s41576-020-0210-7 PMC7752153 32034321

[55] Yang X Zhu Y Chen S Zeng H Guan J Wang Q. Novel allele detection tool benchmark and application with antibody repertoire sequencing dataset. Front Immunol. (2021) 12:739179. doi: 10.3389/fimmu.2021.739179 34764956 PMC8576399

