# Supplementary material for: An allelic atlas of immunoglobulin heavy chain variable regions reveals antibody binding epitope preference resilient to SARS-CoV-2 mutation escape
Source: Front Immunol. 2025 Jan 7;15:1471396. doi: 10.3389/fimmu.2024.1471396 (PMC11746035; doi:10.3389/fimmu.2024.1471396)
Supplement: Supplementary Figure 1 — Summary of IGHV germline sequence (A) Distribution of 111 IGHV families. (B) Comparison of the number of allelic genotypes across 42 antibody families. (C) Comparison of allelic site counts(aa) in each IGHV region. (D) The number of allelic gene loci in CDR1 and CDR2 regions of 26 antibody families. [file DataSheet1.pdf]

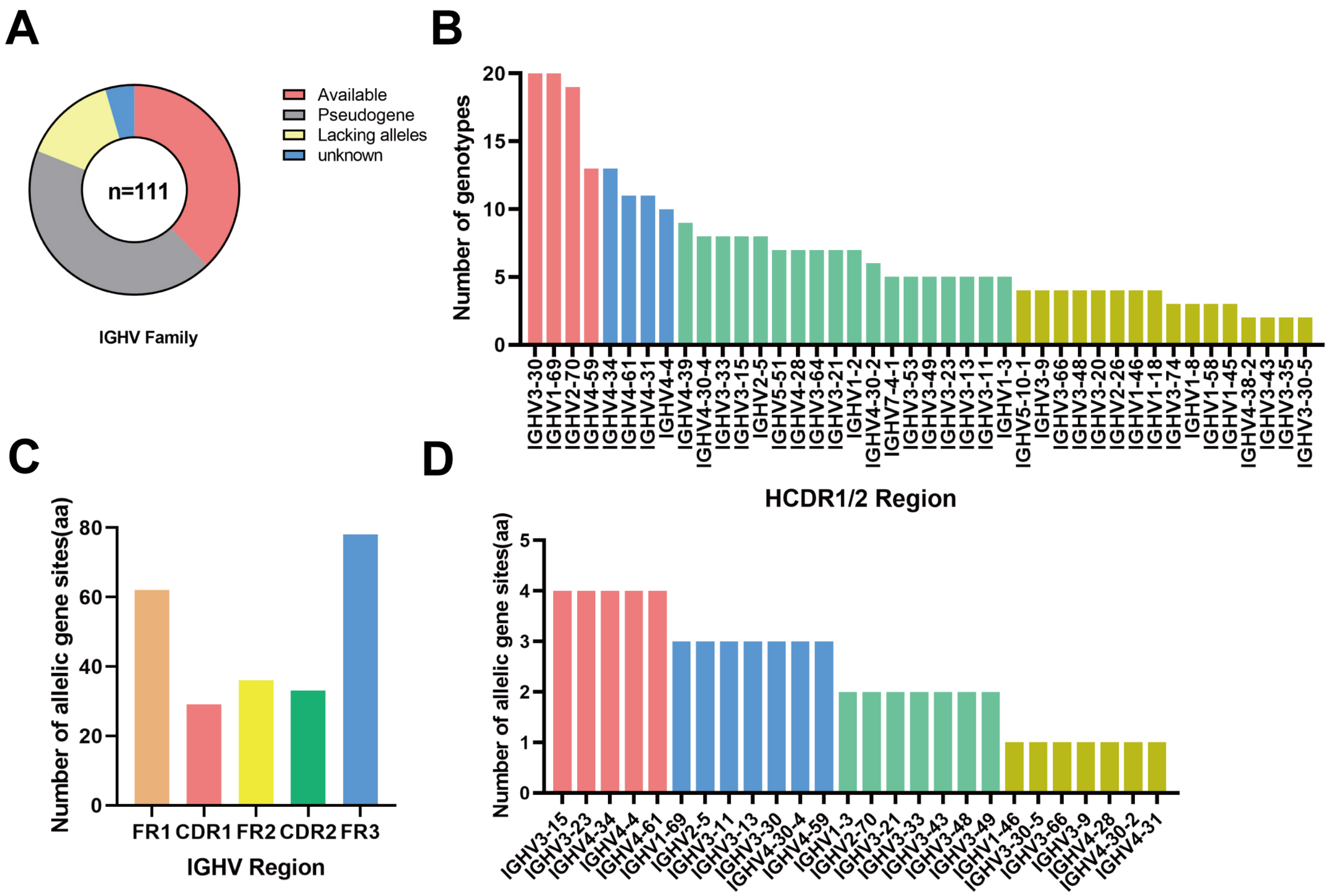
A
n=111
Available
Pseudogene
Lacking alleles
unknown
IGHV Family
B
Number of genotypes
IGHV3-30
IGHV1-69
IGHV2-70
IGHV4-59
IGHV4-34
IGHV4-61
IGHV4-31
IGHV4-4
IGHV4-39
IGHV4-30-4
IGHV3-33
IGHV3-15
IGHV2-5
IGHV5-51
IGHV4-28
IGHV3-64
IGHV3-21
IGHV1-2
IGHV4-30-2
IGHV7-4-1
IGHV3-53
IGHV3-49
IGHV3-23
IGHV3-13
IGHV3-11
IGHV1-3
IGHV5-10-1
IGHV3-9
IGHV3-66
IGHV3-48
IGHV3-20
IGHV2-26
IGHV1-46
IGHV1-18
IGHV3-74
IGHV1-8
IGHV1-58
IGHV1-45
IGHV4-38-2
IGHV3-43
IGHV3-35
IGHV3-30-5
C
Number of allelic gene sites(aa)
FR1
CDR1
FR2
CDR2
FR3
IGHV Region
D
HCDR1/2 Region
Number of allelic gene sites(aa)
IGHV3-15
IGHV3-23
IGHV4-34
IGHV4-4
IGHV4-61
IGHV1-69
IGHV2-5
IGHV3-11
IGHV3-13
IGHV3-30
IGHV4-30-4
IGHV4-59
IGHV1-3
IGHV2-70
IGHV3-21
IGHV3-33
IGHV3-43
IGHV3-48
IGHV3-49
IGHV1-46
IGHV3-30-5
IGHV3-66
IGHV3-9
IGHV4-28
IGHV4-30-2
IGHV4-31

Figure S1

A

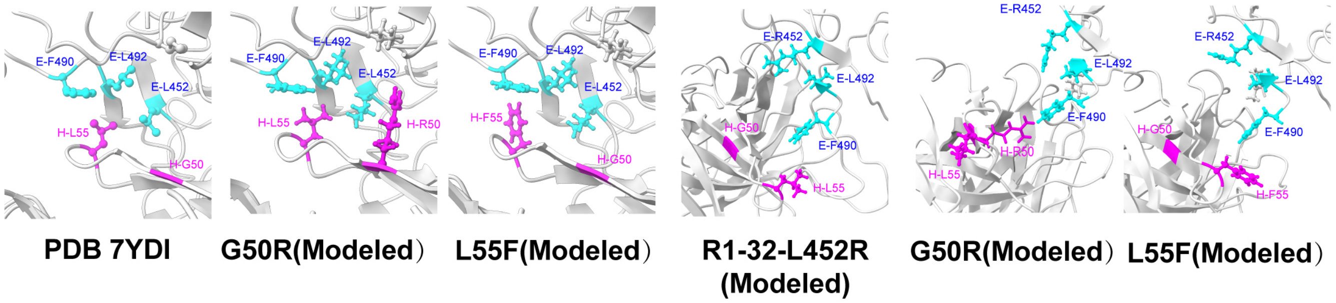

Name	ΔΔG_mut - ΔΔG_wt	Stability
G50R	-2.527	↑
L55F	7.67	↓

Name	ΔΔG_mut - ΔΔG_wt	Stability
G50R	-2.37	↑
L55F	4.25	↓

B

Name	Allele	Epitope	aa50	aa55	J gene(VH)	IGLV	J gene(VL)	HCDR3
R1-32	IGHV1-69*10	E2.2	G	L	IGHJ2	IGLV1-40	IGLJ2	ARENGYSGYGAAANFDL
C091	IGHV1-69*02	E2.2	R	L	IGHJ6	IGLV1-40	IGLJ1	ARDSGYSGYGSTYYMDV
BD56-104	IGHV1-69*02	E2.2	R	L	IGHJ5	IGLV1-40	IGLJ2	ARDVGYSGSGSSNYFDS
BD56-597	IGHV1-69*08	E2.2	R	L	IGHJ5	IGLV1-40	IGLJ2	ARERGYSGYGAAYYFDS
BD56-1711	IGHV1-69*02	E2.2	R	L	IGHJ4	IGLV1-40	IGLJ1	ARGRGYSGYGAAMYCDY
BD56-1834	IGHV1-69*02	E2.2	R	L	IGHJ5	IGLV1-40	IGLJ1	ARVRSIVTINSWWFDP

C

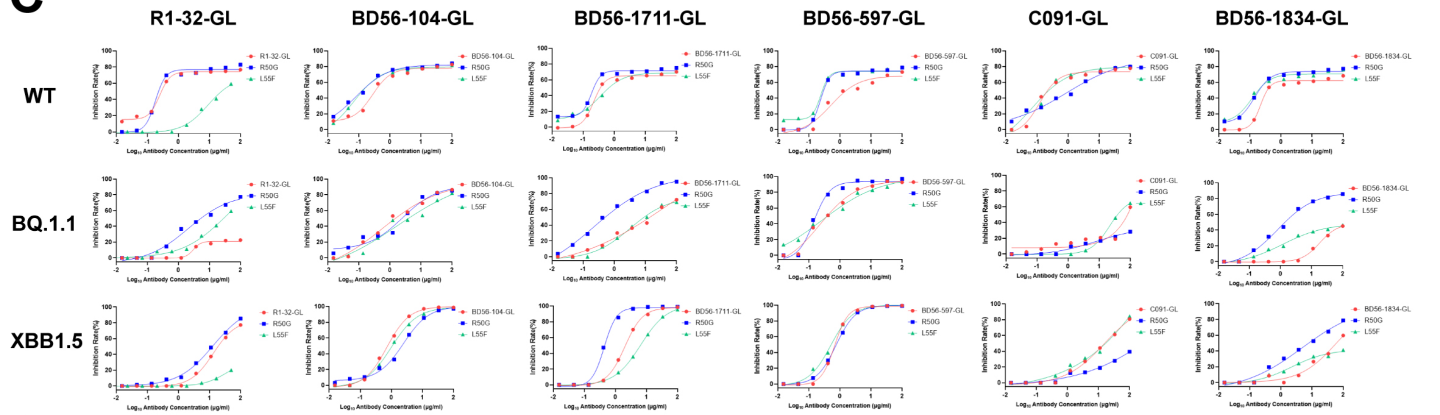

D

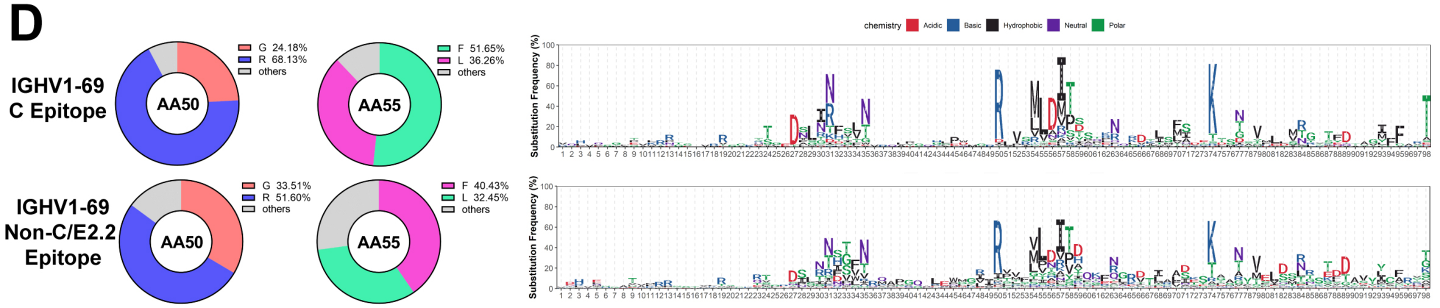

E

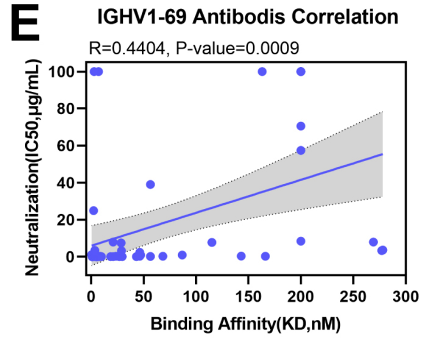

F

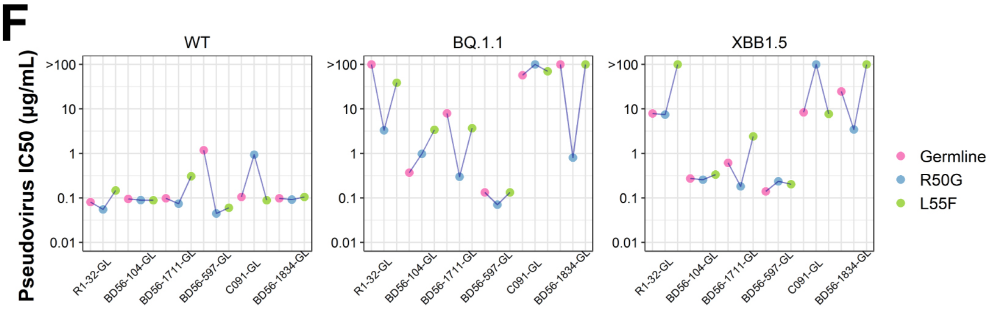

Figure S2

A

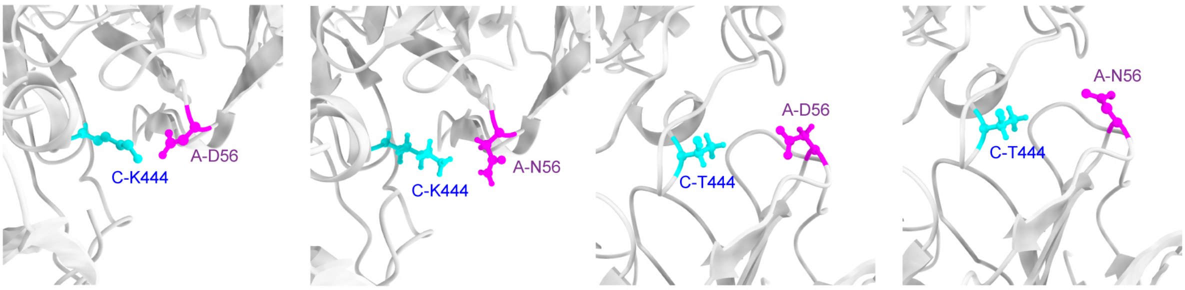

PDB 7MMO | D56N(Modeled) | LY-CoV1404-K444T(Modeled) | D56N(Modeled)

Name	ΔΔG_mut - ΔΔG_wt	Stability
D56N	9.062	↓

Name	ΔΔG_mut - ΔΔG_wt	Stability
D56N	-0.319	↑

B

Name	Allele	Epitope	aa56	J gene(VH)	IGLV	J gene(VL)	HCDR3(aa)
LY-CoV1404	IGHV2-5*02	D2	D	IGHJ1	IGLV2-14	IGLJ2	AHHSISTIFDH
BD56-1290	IGHV2-5*02	D2	D	IGHJ4	IGLV2-14	IGLJ2	GHFTIDMIVGY
BD57-028	IGHV2-5*02	D2	D	IGHJ4	IGLV2-14	IGLJ1	AHHSITTVVDY
XGv-265	IGHV2-5*02	D2	D	IGHJ4	IGLV2-14	IGLJ2	ARHLIPTIFDY
BD56-595	IGHV2-5*02	D2	D	IGHJ4	IGLV2-8	IGLJ1	AQHTIDMILDY
BD56-103	IGHV2-5*01	F1	N	IGHJ4	IGLV3-25	IGLJ2	AHKSRPYSVAYYDDYYFDY
BD55-6297	IGHV2-5*01	F3	N	IGHJ1	IGLV2-14	IGLJ2	AHTMLFEYGDFDY

C

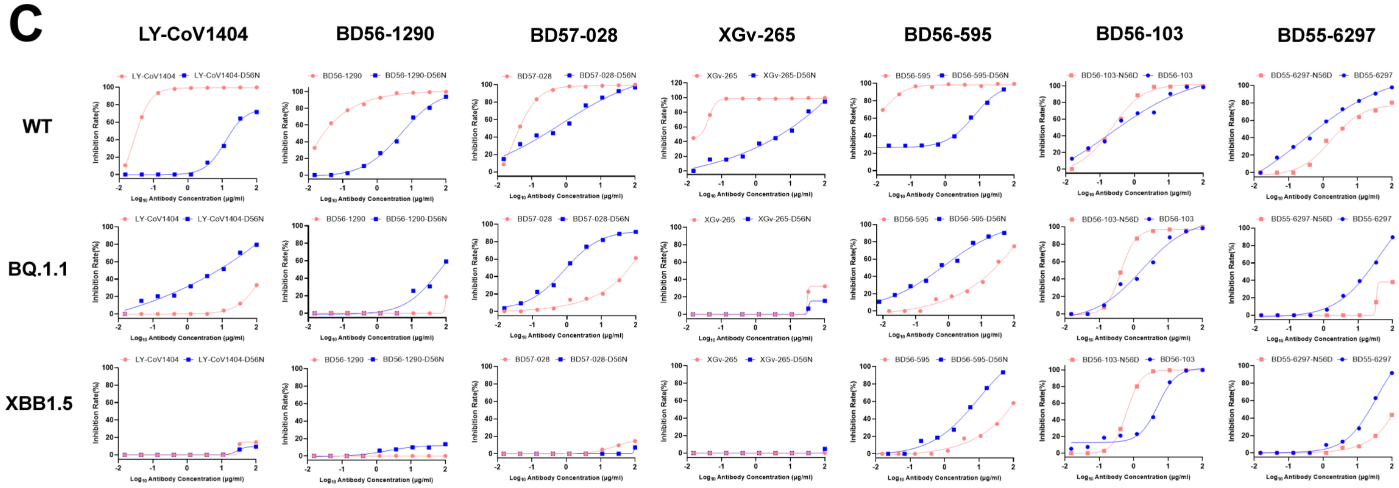

D

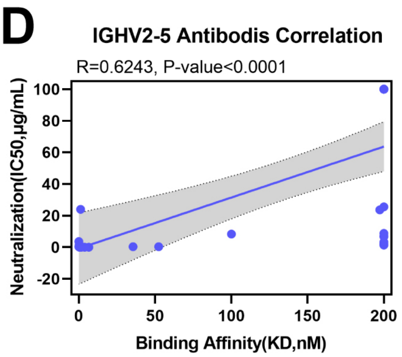

E

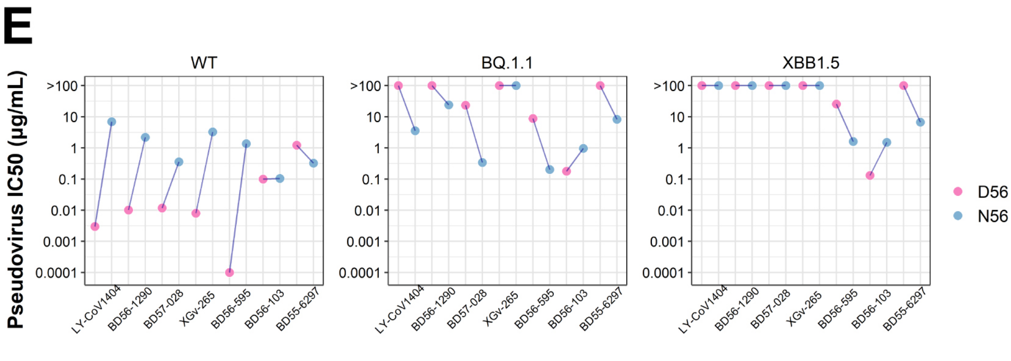

Figure S3

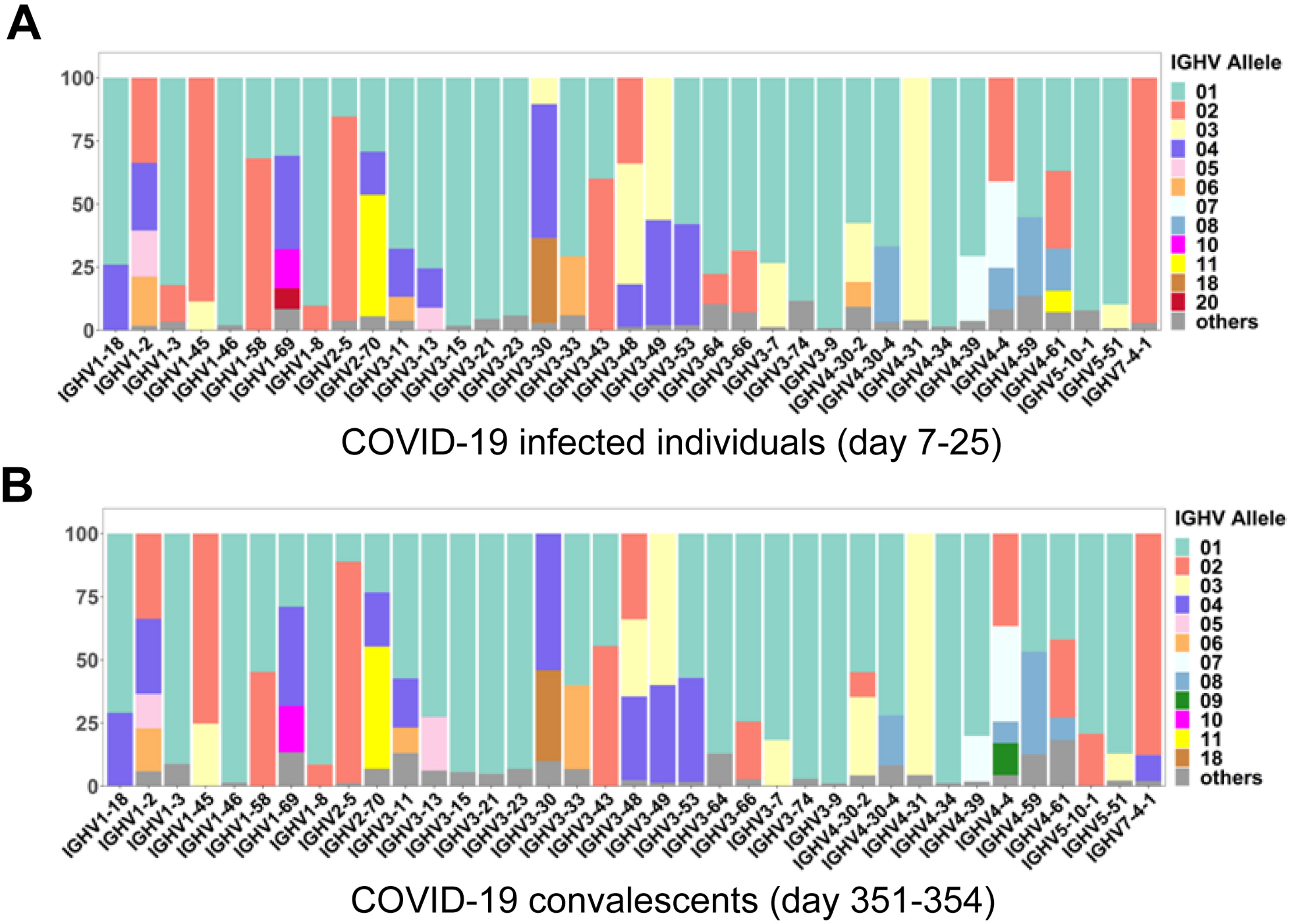
A
B
IGHV Allele
01
02
03
04
05
06
07
08
09
10
11
18
20
others
0
25
50
75
100
IGHV1-18
IGHV1-2
IGHV1-3
IGHV1-45
IGHV1-46
IGHV1-58
IGHV1-69
IGHV1-8
IGHV2-5
IGHV2-70
IGHV3-11
IGHV3-13
IGHV3-15
IGHV3-21
IGHV3-23
IGHV3-30
IGHV3-33
IGHV3-43
IGHV3-48
IGHV3-49
IGHV3-53
IGHV3-64
IGHV3-66
IGHV3-7
IGHV3-74
IGHV3-9
IGHV4-30-2
IGHV4-30-4
IGHV4-31
IGHV4-34
IGHV4-39
IGHV4-4
IGHV4-59
IGHV4-61
IGHV5-10-1
IGHV5-51
IGHV7-4-1
COVID-19 infected individuals (day 7-25)
COVID-19 convalescents (day 351-354)

Figure S4
