# Supplementary material for: An allelic atlas of immunoglobulin heavy chain variable regions reveals antibody binding epitope preference resilient to SARS-CoV-2 mutation escape
Source: Front Immunol. 2025 Jan 7;15:1471396. doi: 10.3389/fimmu.2024.1471396 (PMC11746035; doi:10.3389/fimmu.2024.1471396)
Supplement: Supplementary file 3 [file Table2.docx]

| **IGHV1-69** | **RBD-WT** | | | **RBD- BQ1.1** | | | **RBD- XBB** | | |
| --- | --- | --- | --- | --- | --- | --- | --- | --- | --- |
|  | *k*_on_ (M^−1^ s^−1^) | *k*_off_ (s^−1^) | *K*_D_(nM) | *k*_on_ (M^−1^ s^−1^) | *k*_off_ (s^−1^) | *K*_D_(nM) | *k*_on_ (M^−1^ s^−1^) | *k*_off_ (s^−1^) | *K*_D_(nM) |
| C091-Germline | 3.50×10^5^ | 1.50×10^-3^ | 4.29 | - | - | no binding | 1.24×10^5^ | 1.53×10^-3^ | 92.5 |
| C091-R50G | 1.82×10^5^ | 8.58×10^-3^ | 47.1 | - | - | no binding | - | - | no binding |
| C091-L55F | 2.62×10^5^ | 4.85×10^-3^ | 18.5 | - | - | no binding | 9.23×10^4^ | 2.68×10^-2^ | 290.6 |
|  |  |  |  |  |  |  |  |  |  |
| BD-597- Germline | 4.07×10^5^ | 1.15×10^-4^ | 0.28 | 2.37×10^5^ | 1.69×10^-3^ | 7.11 | 6.56×10^5^ | 2.20×10^-4^ | 0.34 |
| BD-597-R50G | 1.53×10^6^ | 1.24×10^-4^ | 0.08 | 8.30×10^5^ | 3.33×10^-4^ | 0.4 | 1.05×10^6^ | 1.48×10^-3^ | 1.41 |
| BD-597-L55F | 2.50×10^5^ | 1.32×10^-3^ | 5.28 | 1.98×10^5^ | 5.84×10^-3^ | 29.5 | 7.26×10^5^ | 7.51×10^-4^ | 1.03 |
|  |  |  |  |  |  |  |  |  |  |
| R1-32- Germline | 7.19×10^5^ | 1.11×10^-3^ | 1.54 | - | - | no binding | 3.40×10^5^ | 7.05×10^-3^ | 20.8 |
| R1-32-R50G | 8.42×10^5^ | 6.61×10^-4^ | 0.78 | 2.71×10^5^ | 7.80×10^-3^ | 28.8 | 3.39×10^5^ | 9.66×10^-3^ | 28.5 |
| R1-32-L55F | 6.05×10^5^ | 3.73×10^-3^ | 6.17 | 3.94×10^5^ | 2.23×10^-2^ | 56.5 | - | - | no binding |
|  |  |  |  |  |  |  |  |  |  |
| BD56-104-Germline | 2.58×10^5^ | 9.99×10^-4^ | 3.87 | 9.17×10^4^ | 1.31×10^-2^ | 142.6 | 8.75×10^4^ | 1.59×10^-3^ | 8.34 |
| BD56-104-R50G | 4.47×10^5^ | 4.99×10^-4^ | 1.12 | 2.36×10^5^ | 2.05×10^-2^ | 86.8 | 1.69×10^5^ | 9.50×10^-3^ | 68.3 |
| BD56-104-L55F | 4.21×10^5^ | 2.76×10^-3^ | 6.55 | 1.08×10^5^ | 2.99×10^-2^ | 277 | 1.99×10^5^ | 9.18×10^-3^ | 46.2 |
|  |  |  |  |  |  |  |  |  |  |
| BD56-1711- Germline | 1.19×10^5^ | 3.55×10^-3^ | 29.9 | 4.16×10^4^ | 1.10×10^-2^ | 263.8 | 8.75×10^4^ | 1.59×10^-3^ | 18.12 |
| BD56-1711-R50G | 2.17×10^5^ | 4.54×10^-3^ | 20.9 | 1.64×10^5^ | 3.87×10^-3^ | 23.6 | 1.69×10^5^ | 9.50×10^-3^ | 56.38 |
| BD56-1711-L55F | 1.41×10^5^ | 2.34×10^-2^ | 165.5 | 7.93×10^4^ | 2.21×10^-2^ | 278.3 | 1.99×10^5^ | 9.18×10^-3^ | 46.15 |
|  |  |  |  |  |  |  |  |  |  |
| BD56-1834- Germline | 3.98×10^5^ | 2.56×10^-3^ | 6.44 | 1.69×10^5^ | 1.88×10^-3^ | 110.9 | 2.34×10^5^ | 1.04×10^-3^ | 4.43 |
| BD56-1834-R50G | 4.25×10^5^ | 2.70×10^-3^ | 6.35 | 6.95×10^5^ | 2.69×10^-2^ | 38.8 | 8.76×10^5^ | 6.27×10^-2^ | 71.55 |
| BD56-1834-L55F | 4.21×10^5^ | 1.81×10^-2^ | 43.0 | 1.10×10^6^ | 1.64×10^-2^ | 14.9 | 5.20×10^5^ | 8.50×10^-2^ | 163.3 |
|  |  |  |  |  |  |  |  |  |  |
|  |  |  |  |  |  |  |  |  |  |
