# Supplementary material for: An allelic atlas of immunoglobulin heavy chain variable regions reveals antibody binding epitope preference resilient to SARS-CoV-2 mutation escape
Source: Front Immunol. 2025 Jan 7;15:1471396. doi: 10.3389/fimmu.2024.1471396 (PMC11746035; doi:10.3389/fimmu.2024.1471396)
Supplement: Supplementary file 4 [file Table3.docx]

| **IGHV2-5** | **RBD-WT** | | | **RBD-BQ1.1** | | | **RBD-XBB** | | |
| --- | --- | --- | --- | --- | --- | --- | --- | --- | --- |
|  | *k*_on_ (M^−1^ s^−1^) | *k*_off_ (s^−1^) | *K*_D_(nM) | *k*_on_ (M^−1^ s^−1^) | *k*_off_ (s^−1^) | *K*_D_(nM) | *k*_on_ (M^−1^ s^−1^) | *k*_off_ (s^−1^) | *K*_D_(nM) |
| **LYCoV-1404** | 3.47×10^5^ | 1.30×10^-3^ | 3.75 | - | - | no binding | - | - | no binding |
| **LYCoV-1404-D56N** | - | - | no binding | - | - | no binding | - | - | no binding |
| **BD56-1290** | 2.35×10^5^ | 1.52×10^-3^ | 6.44 | - | - | no binding | - | - | no binding |
| **BD56-1290-D56N** | - | - | no binding | 2.04×10^5^ | 2.37×10^-4^ | 1.16 | - | - | no binding |
| **BD57-028** | 2.32×10^5^ | 2.03×10^-4^ | 0.87 | 1.60×10^5^ | 3.16×10^-2^ | 197 | - | - | no binding |
| **BD57-028-D56N** | 1.34×10^5^ | 7.02×10^-3^ | 52.3 | 1.72×10^5^ | 6.14×10^-3^ | 35.6 | - | - | no binding |
| **XGv-265** | 2.46×10^5^ | 1.01×10^-4^ | 0.41 | - | - | no binding | - | - | no binding |
| **XGv-265-D56N** | - | - | no binding | - | - | no binding | - | - | no binding |
| **BD56-595** | 1.52×10^5^ | 1.55×10^-3^ | 10.2 | - | - | no binding | - | - | no binding |
| **BD56-595-D56N** | - | - | no binding | 1.81×10^5^ | 1.02×10^-1^ | 1.82 | - | - | no binding |
| **BD56-103** | 5.57×10^5^ | <1.0×10^-7^ | <0.001 | 7.33×10^5^ | <1.0×10^-7^ | <0.001 | 8.14×10^5^ | <1.0×10^-7^ | <0.001 |
| **BD56-103-D56N** | 5.70×10^5^ | <1.0×10^-7^ | <0.001 | 7.40×10^5^ | <1.0×10^-7^ | <0.001 | 7.25×10^5^ | <1.0×10^-7^ | <0.001 |
| **BD55-6279** | 1.23×10^5^ | 1.0×10^-7^ | 0.06 | - | - | no binding | - | - | no binding |
| **BD55-6279-N56D** | 1.06×10^5^ | 6.64×10^-5^ | <0.001 | - | - | no binding | - | - | no binding |
|  |  |  |  |  |  |  |  |  |  |
